# Inhibition of RNA-binding proteins enhances immunotherapy in ovarian cancer

**DOI:** 10.1038/s41392-025-02515-1

**Published:** 2025-12-25

**Authors:** Nadine Bley, Alexander Rausch, Simon Müller, Theresa Simon, Markus Glaß, Danny Misiak, Laura Schian, Lara Meret Peters, Mohammad Dipto, Ali Hmedat, Bianca Busch, Annekatrin Schott, Marcell Lederer, Alice Wedler, Robin Benedikt Rolnik, Hend Elrewany, Ehab Ghazy, Wolfgang Sippl, Martina Vetter, Markus Wallwiener, Stefan Hüttelmaier

**Affiliations:** 1 Institute of Molecular Medicine, Section for Molecular Cell Biology, Faculty of Medicine, Martin Luther University Halle-Wittenberg, Halle, Germany; 2 New York Genome Center, New York, NY USA; 3 Department of Biology, New York University, New York, NY USA; 4 Department of Pharmaceutics and Pharmaceutical Technology, Faculty of Pharmacy, Yarmouk University, Irbid, Jordan; 5 Department of Medicinal Chemistry, Institute of Pharmacy, Martin Luther University Halle-Wittenberg, Halle, Germany; 6 Department of Gynecology, University Hospital, Martin Luther University Halle-Wittenberg, Halle, Germany

**Keywords:** Tumour immunology, Gynaecological cancer

## Abstract

High-grade serous ovarian cancer (HGSC) accounts for more than 70% of ovarian cancer-related deaths, yet therapeutic progress remains stagnant. Among the four molecular subtypes reported for HGSC, the C5 subtype is distinguished by high proliferation and immune evasion with an unfavorable MHC-I/*PD-L1* ratio. However, the molecular drivers of this immune desert state remain largely undefined. Here, we identify RNA-binding proteins (RBPs) as key regulators of immune evasion in C5-HGSC through integrated single-cell and bulk RNA sequencing. We perform a targeted loss-of-function screen in C5-like cell models and find *IGF2BP1* as a central mediator of immune evasion in vitro and in vivo. Mechanistically, *IGF2BP1* abrogates interferon-gamma signaling by accelerating *IRF1* protein degradation, thereby suppressing MHC-I presentation. We also discover that *IGF2BP1* decouples *PD-L1* expression from *IRF1*-dependent transcription and reshapes the immune receptor landscape to limit immune cell infiltration and T cell activation. Therapeutically, the small-molecule BTYNB effectively inhibits *IGF2BP1* and synergizes with PD-1 blockade to overcome immune evasion in vivo. Multi-spectral imaging confirms these findings in human HGSC tissues and highlights the role of oncofetal RBPs as molecular drivers of the C5-HGSC subtype. This subtype-wide survey uncovers a previously unrecognized RBP–interferon regulatory axis and establishes RBP inhibition as a therapeutic strategy to enhance immune checkpoint therapy in immunologically cold ovarian tumors.

## Introduction

HGSC accounts for the majority (>70%) of ovarian cancer-associated deaths.^1^ This adverse outcome largely arises from late symptom onset and diagnosis at advanced stages characterized by extensive peritoneal spread. Debulking surgery and standard chemotherapy remain the major determinants of therapy outcomes, and despite temporary responses, recurrence and therapy resistance are almost inevitable. Pathological and genomic analyses, along with studies using ovarian cancer models, provide strong evidence that most HGSCs originate from secretory epithelial cells of the distal fallopian tube.^2^ The mutational burden is rare in HGSC with the exception of *TP53* (>95%) and, less prominently, *BRCA1* and *BRCA2* inactivation.^3,4^ However, due to the deficiency of homologous repair (HR) pathways in approximately 50% of cases, HGSC presents an outstanding example of C-class malignancies, defined by extensive DNA copy number variations, including recurrent gains at chromosome 17q and *CCNE1* amplification.^4^ HR deficiency underpins sensitivity to platinum-based therapies and supports the therapeutic potential of poly(ADP-ribose) polymerase (PARP) inhibitors.^1,4^ In contrast, HR-proficient or *CCNE1*-amplified tumors show limited response to these therapies.^5^ To address therapeutic resistance observed with monotherapies, current approaches, and clinical trials are exploring combination therapies targeting PARP, angiogenesis, kinases, and immune checkpoints.^6^ However, immune checkpoint inhibition (ICI) largely failed to improve patient survival due to the heterogeneous immune landscape of HGSC tumors and the lack of robust predictive biomarkers for patient response.^7^ This lack of durable benefit from immunotherapy stands in stark contrast to success of ICI in other solid tumors. Therefore, identifying druggable molecular determinants of immune evasion is essential to improve therapeutic efficacy in HGSC.

Efforts to overcome these challenges have identified distinct cellular and molecular subtypes based on transcriptome signatures, clinical outcomes, cellular composition, and tumor microenvironment activity. According to the immune cell landscape, these classifications distinguish immune cell-infiltrated, excluded and desert tumors.^8^ Molecular subtypes are defined as C1 (mesenchymal), C2 (immune responsive), C4 (differentiated), and C5 (proliferative and immune-evasive). Notably, C1 and C5 subtypes are associated with the most adverse patient outcome.^4,9^ In accord, C5-type tumors are characterized by reduced immune, particularly T cell, infiltration and a dedifferentiated, mesenchymal, stem cell-like gene expression. These features are indicative of a highly plastic, therapy-resistant cell state that contributes to immune suppression and tumor persistence. However, this phenotype is not associated with a specific mutational pattern, implying that its regulation likely occurs at transcriptional or posttranscriptional levels.^10^ However, the molecular basis of such regulatory programs active in C5 tumors remains poorly understood, impeding the development of strategies to re-activate immune cells and enhance ICI efficacy.^10,11^

To identify molecular players specific to the C5 subtype, we analyzed six independent bulk-seq and single-cell RNA-seq (scRNA-seq) datasets, integrating tumor-intrinsic and microenvironmental gene expression programs. This multi-cohort analysis revealed a consistent enrichment of RBP-driven regulatory modules associated with C5 properties, suggesting that RBPs may constitute a critical and previously underappreciated layer of immune regulation in HGSC. RNA-binding proteins (RBPs) are critical regulators of gene expression and orchestrate the fate of (m)RNA in various biological aspects of development, homeostasis, and disease.^12^ In cancer, RBPs often exhibit aberrant expression and promote tumorigenesis by modulating key cancer hallmarks such as cellular proliferation and immune evasion.^13,14^ RBPs can support cancer aggressiveness by a “by-the-numbers” principle, since they represent oncogenic protein nodes that can modulate multiple signaling pathways simultaneously.^15^ Previously considered undruggable, oncofetal RBPs enriched in stem cells—such as IGF2 mRNA binding proteins (*IGF2BP1-3*), LIN-28 RNA binding posttranscriptional regulators (*LIN28A/B*) and Musashi proteins (*MSI1/2*)—have recently emerged as promising strategies for targeted therapies.^16^

In this study, we identify eight oncofetal RBPs as molecular drivers of the immune-suppressed C5-subtype in HGSC and demonstrate their role in promoting immune evasion through a targeted loss-of-function screen. As proof-of-concept, we show that the lead candidate *IGF2BP1* uncouples *PD-L1* from interferon-dependent signaling to modulate the immune receptor landscape and evade T cell-mediated anti-tumor immunity. Targeting *IGF2BP1* by small molecules reverses immune evasion and enhances PD-1-directed ICI efficacy in vitro and in vivo. Our findings establish RBPs as central regulators of the C5-specific immune-suppressed state and reveal an unrecognized posttranscriptional mechanism underlying immune checkpoint resistance in HGSC. By elucidating how IGF2BP1 and related oncofetal RBPs coordinate immune evasion, this work expands current concepts of tumor–immune interactions beyond transcriptional control. Moreover, it highlights the therapeutic potential of targeting RBP-dependent regulatory networks to sensitize immune-desert HGSC subtypes to immunotherapy. Collectively, our study provides a conceptual framework for integrating posttranscriptional control into precision oncology approaches and suggests that modulation of RBP activity could represent a new avenue to overcome the intrinsic immune resistance of HGSC.

## Results

### Oncofetal RBPs distinguish C5-HGSC

**Fig. 1:**
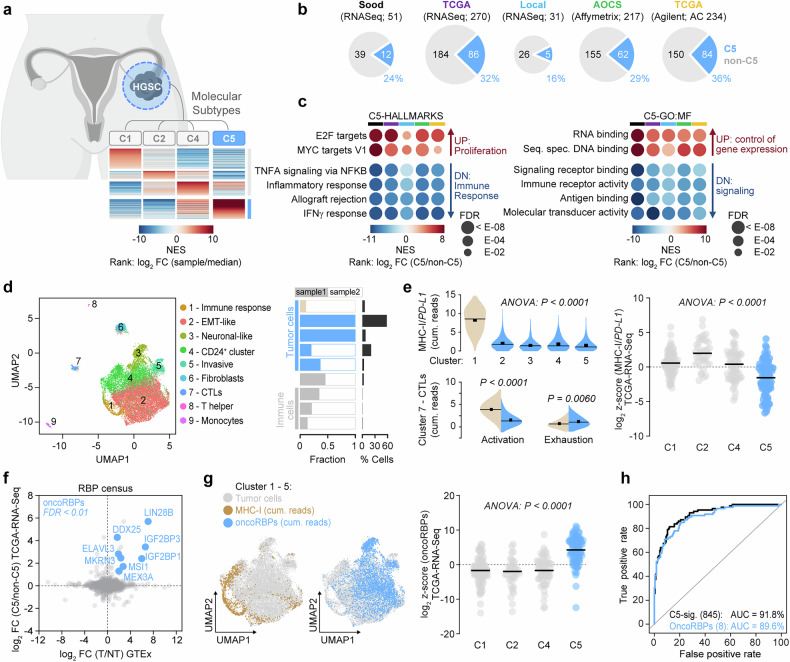
C5 tumors exhibit low immune cell infiltration and elevated RBP expression. **a** Schematic illustrating the classification of HGSC tumor cohorts into molecular subtypes (C1, C2, C4, C5) using GSEA and subtype signatures established by Tothill et al.^9^ **b** Pie charts displaying the frequencies (%) and numbers of C5 (blue) and non-C5 (gray) cases in the indicated HGSC-datasets. The analysis platforms and the total number of unambiguously classified cases are provided. TCGA-AC includes additional cases (AC) absent from the TCGA-RNA-Seq cohort. **c** Bubble chart illustrating enriched (UP) and depleted (DN) gene sets in C5-HGSCs, associated with cancer hallmarks or gene ontology – molecular function (GO: MF) determined by GSEA. Color bars correspond to datasets shown in (**b**): Sood (black), TCGA-OV-RNA-Seq (purple), Local (blue), AOCS (green), and TCGA-AC (yellow). **d** scRNA-Seq analysis of HGSC samples. UMAP of 19,151 cells (left) indicates identified tumor and stromal cell clusters. Sample distribution (fraction) and total cluster content (% cells) are presented as bar plots. **e** Violin blots (left) show cumulative scRNA-seq reads for MHC-I/*PD-L1* ratios, CTL activation (*IFNG*, *GZMB*, *CD69*), and exhaustion (*CXCL13*, *HAVCR2*, *LAYN*) associated with immunologically warm (yellow) and cold (blue) cluster. Scatter dot plot (right) indicates reduced MHC-I/*PD-L1* ratios of C5-HGSCs. One-way ANOVA testing was used to determine statistical significance. **f** Scatter plot of the RBP census.^22^ The top-eight RBPs (oncoRBPs) dysregulated across datasets are highlighted in blue. **g** UMAPs (left) indicate cumulative scRNA-seq reads for MHC-I (yellow) or oncoRBPs (blue). Scatter dot plot (right) indicates oncoRBP expression in HGSC subtypes. **h** ROC analysis evaluating the oncoRBP signature (blue) as a classifier for C5 tumors. The C5 signature^9^ (black) served as a control for comparison

To identify molecular drivers of the proliferative and immune-evasive C5 subtype in HGSC, we analyzed gene expression signatures from five independent HGSC cohorts.^4,9,17,18^ We classified tumors into C5 and non-C5 tumor clusters using gene set enrichment analysis (GSEA) (Fig. 1a, b and Supplementary Table 1). Consistent with prior studies,^4,9^ GSEA confirmed that C5 tumors are enriched for pro-proliferative gene sets, such as E2F target genes, while immune signaling pathways, including IFNγ response, are downregulated (Fig. 1c and Supplementary Table 2). C5 tumors also showed reduced immune cell infiltration, notably of cytotoxic T cells (CTLs), macrophages, and monocytes, with CTL abundance linked to prognostic significance (Supplementary Fig. S1a). We also found that immune suppression was independent of mutational load, as C5 and non-C5 tumors showed comparable mutational burden and copy number variations (CNVs) (Supplementary Fig. S1b).

Next, we performed scRNA-seq and identified five distinct tumor cell clusters within C5-HGSC: immune-responsive, EMT-like, neuronal-like, *CD24*^+^ stem cell-like, and invasive (Fig. 1d and Supplementary Tables 3 and 4). The invasive cluster showed enhanced Rho-GTPase and FAK signaling, while the EMT-like cluster was enriched for SMAD/TGFβ pathways (Supplementary Fig. S1c). The *CD24*^+^ and neuronal-like clusters, representing cancer stem cells, were enriched for proliferative gene sets, DNA repair mechanisms, and β−catenin signaling. Notably, *CD24*^+^ stem cells and the neural circuit have also recently been linked to immune evasion in ovarian cancer.^19,20^ RNA metabolism and translation were upregulated in *CD24*^+^ or EMT tumor cell clusters, respectively, whereas the immune-responsive cluster uniquely showed enhanced interferon and interleukin signaling. Except for this cluster, all tumor cell clusters demonstrated low MHC-I/*PD-L1* ratios, indicative of an immunosuppressive tumor microenvironment (Fig. 1e). Bulk RNA-seq of C5-HGSC patient samples further corroborated this, with higher MHC-I/*PD-L1* ratios associated with improved prognosis (Fig. 1e and Supplementary Fig. S1d). Correspondingly, CTLs from patients with low MHC-I/*PD-L1* ratios showed reduced activation and elevated exhaustion markers.^21^ These findings indicate that an unfavorable MHC-I/*PD-L1* ratio contributes to the immunologically cold phenotype of C5-HGSC.

The downregulated immune response signatures in C5-HGSC coincided with a previously unreported upregulation of genes with RNA-binding capacity (Fig. 1c and Supplementary Table 5). We further analyzed the expression of individual RNA-binding proteins (RBPs) from the RBP census^22^ and found a significant upregulation of eight canonical RBPs — *DDX25*, *ELAVL3*, *IGF2BP1*, *IGF2BP3*, *LIN28B*, *MEX3A*, *MKRN3*, and *MSI1*—in C5 tumors compared to non-C5 tumors or normal tissue (Fig. 1f). These RBPs were enriched across all tumor cell clusters except the immune-responsive cluster and consistently upregulated across all five tumor cohorts, while correlating with poor survival outcomes (Fig. 1g and Supplementary Fig. S1e). Notably, these RBPs exhibited an oncofetal expression pattern, with expression largely restricted to tumor cells and cancer-associated fibroblasts (CAFs) but minimal in immune cells (Supplementary Fig. S1f, g).

Several of these RBPs are implicated in immune evasion. *MSI1*, *LIN28B*, and IGF2BPs promote proliferation, stemness, and immune evasion in solid cancers,^23–28^ while *DDX25* and *MEX3A* inhibit immune responses by suppressing viral defenses or promoting *RIG-I* degradation, respectively.^29,30^ *ELAVL3*, together with *ALK* and *SOX* transcription factors, enhances the neural circuit,^31^ which is linked to tumor aggressiveness and immune evasion.^20^ In HGSC, the top-eight RBP signature correlated with an unfavorable MHC-I/*PD-L1* ratio (Supplementary Fig. S1h), effectively distinguished C5 from non-C5 tumors (Fig. 1g), and classified C5 tumors with ~90% AUC accuracy (Fig. 1h). Remarkably, this classification mirrored the performance of a previously reported C5 gene panel of over 800 genes, despite the latter including only two RBPs (*MEX3A* and *LIN28B*) from our top-eight signature. These findings suggest that C5-specific oncofetal RBPs (oncoRBPs) may drive immune evasion by directly downregulating MHC-I and upregulating *PD-L1* expression in HGSC.

### OncoRBPs promote immune evasion

To evaluate the role of candidate oncoRBPs in immune evasion, we first assessed *TP53*-mutated C5-like cell models from the Cancer Cell Line Encyclopedia (CCLE), since *TP53* mutation is a hallmark of HGSC that distinguishes it from other ovarian cancer histological subtypes.^3^ Among these, we identified ES-2, OVCAR-8, along with endometrial cancer-derived TOV112D, as top-ranking models, characterized by high proliferative capacity and strong immune suppression (Supplementary Fig. S2a). Although ES-2 cells were originally described as clear cell ovarian carcinoma–derived, they were considered HGSC-like in line with previous reports due to their mutational and transcriptomic profile.^32–34^

**Fig. 2:**
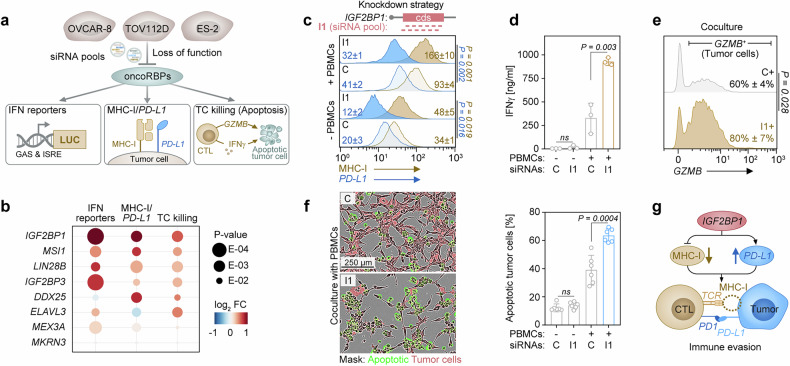
OncoRBPs promote immune evasion. **a** Schematic of the screen targeting the top-eight dysregulated oncoRBPs in HGSC using three C5-like cell lines and pools of 5 siRNAs per RBP to determine: interferon-dependent transcription via GAS and IRSE luciferase reporters (left), MHC-I/*PD-L1* ratios (middle), and T cell-mediated tumor cell killing (TC killing) by caspase 3/7 activity (right). **b** Bubble chart summarizes results of the siRNA screen (**a**) as average log_2_ FC of all three cell lines. An unpaired two-tailed t test was used to determined statistical significance. **c–f** ES-2 cells were transfected with a pool of 12 *IGF2BP1*-directed siRNAs (I1; schematic in **c**) or control siRNAs (C) and cultured in the presence (+) or absence (-) of PBMCs. Histograms indicate MHC-I and *PD-L1* presentation (**c**). Changes in IFNγ secretion determined by ELISA are shown as a bar diagram (**d**). Histograms indicate granzyme B (*GZMB*) staining within tumor cells (**e**). Numbers represent MFI (**c**), % positive cells and standard deviation (s.d.) of three independent experiments per condition. Representative images were overlaid with segmentation masks (f, left) and quantifications of caspase-3/7 activities are shown as a bar diagram (**f**, right). Statistical significance was determined by an unpaired two-tailed t test. **g** Schematic illustrates alterations of MHC-I and *PD-L1* presentation directed by *IGF2BP1* to promote evasion from T cell-mediated tumor cell killing

We assessed the impact of the eight candidate oncoRBPs on immune phenotypes dysregulated in C5-tumors by siRNA-pool-directed depletion. We monitored: (1) interferon-dependent gene expression using ISRE and GAS luciferase reporters; (2) MHC-I/*PD-L1* surface expression ratios; and (3) tumor cell killing by HLA-matched peripheral blood mononuclear cells (PBMCs), measured via caspase-3/7 activity (Fig. 2a). Across the three C5-like models, log_2_ fold-changes of these parameters ranked the oncoRBPs by their immune evasion potential (Fig. 2b and Supplementary Table 6). The depletion of all oncoRBPs except *MKRN3* altered at least two phenotypic readouts, with *IGF2BP1*, *MSI1*, and *LIN28B* showing the most consistent and pronounced effects.

Since IGF2BP1 had the most significant impact in our screen, we investigated this RBP in further detail. Using a pool of 12 siRNAs to minimize off-target effects^35^ (Fig. 2c; I1 siRNA pool), and alternatively an siRNA targeting the 3′UTR of IGF2BP1 (Supplementary Fig. S2b; 3p siRNA). We found that *IGF2BP1* depletion improved the immune receptor landscape by increasing MHC-I presentation while reducing *PD-L1* levels (Fig. 2c and Supplementary Fig. S2b). These changes were reversed by *IGF2BP1* re-expression or dose-dependent overexpression (Supplementary Fig. S2c, d). MHC-I/*PD-L1* alterations persisted or intensified upon exposure to HLA-matched PBMCs or IFNγ-treatment (Fig. 2c and Supplementary Fig. S2e). Accordingly, *IGF2BP1* depletion enhanced T cell activation and tumor cell killing in the presence of HLA-matched PBMCs, as evidenced by increased IFNγ secretion (Fig. 2d), elevated granzyme B (*GZMB*) levels within tumor cells (Fig. 2e), and enhanced caspase-3/7 activity (Fig. 2f and Supplementary Fig. S2b for an alternative siRNA). Notably, IGF2BP1-dependent alterations in MHC-I and *PD-L1* presentation were retained even in more immune-responsive HGSC cell lines such as COV318, which express IGF2BP1 (Supplementary Fig. S2f), albeit with more modest effects due to their already high basal MHC-I expression. Moreover, this *IGF2BP1*-mediated immune-evasive program was conserved in murine ID8 cells. In these, *Igf2bp1* expression is induced following *Trp53* deletion and *Igf2bp1* knockout increased the MHC-I/PD-L1 ratio (Supplementary Fig. S2g). These findings reveal RBP-driven mechanisms of immune evasion in C5-HGSC characterized by disrupted MHC-I and *PD-L1* regulation. This leads to an unfavorable immune-receptor landscape on tumor cells and impairment of T cell responses (Fig. 2g).

### IGF2BP1 promotes immune evasion in vivo

**Fig. 3:**
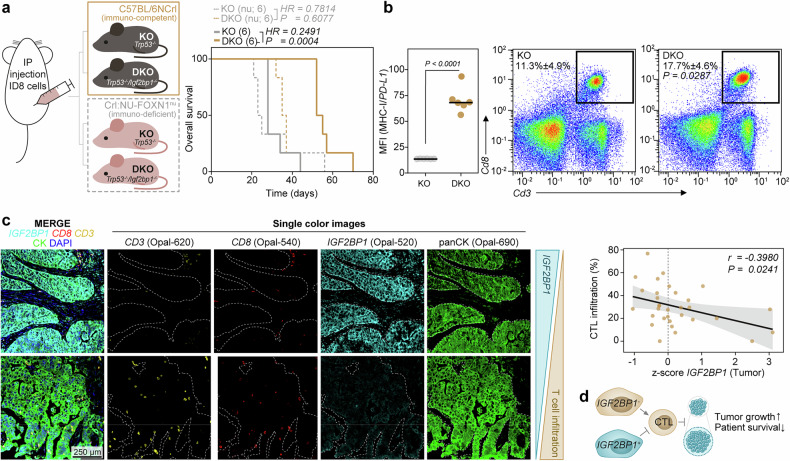
*IGF2BP1* prevents T cell infiltration in murine and human models. **a** Schematic representation of the experimental setup for an intraperitoneal (IP) ovarian cancer model using immuno-competent (C57BL/6NCrl) or immuno-deficient (Crl:NU-FOXN1^nu^) mice injected with iRFP-labeled ID8-*Trp53*-KO^36^ or ID8-*Trp53/Igf2bp1*-DKO cells. Tumor growth was monitored via iRFP fluorescence, and the development of palpable ascites served as the termination criterion. Survival data are presented as a Kaplan–Meier plot, with solid lines representing immuno-competent mice and dashed lines representing immuno-deficient mice using 6 mice per condition. Hazard ratio (HR) and statistical significance were determined by a logrank test. **b** Flow cytometry analyses of ascitic fluid were used to assess the MHC-I/*PD-L1* ratio on iRFP-positive tumor cells (left). Density plots illustrate the proportion of *Cd8*^+^/*Cd3*^+^-CTLs in the ascitic fluid. The average CTL-percentage and s.d. of 6 mice is depicted as numbers. Statistical significance was determined by an unpaired two-tailed t test. **c** Multispectral imaging of a human HGSC-TMA, sequentially stained with the indicated antibodies using the Opal-7 system. Representative images with high (upper panel) or low IGF2BP1 expression (lower panel) are shown as overlay (merge) and single-color images. Single-cell segmentation and classification were performed using QuPath.^66^ Pearson correlation analysis was applied to evaluate the relationship between *IGF2BP1*-expressing, cytokeratin-positive (panCK) tumor cells and infiltrating *CD8*^+^/*CD3*^+^-CTLs. **d** Schematic depiction of *IGF2BP1*-mediated suppression of T cell activation and infiltration

To assess *IGF2BP1*’s role in tumor growth and immune evasion in vivo, we used ID8 cells—a syngeneic murine model of HGSC with CRISPR-Cas9-mediated knockout of *Trp53*^36^—that retain *Igf2bp1*-driven immune evasion in vitro (Supplementary Fig. S2g). To separate immune-mediated effects from the previously reported growth-promoting functions of *IGF2BP1,*^37–39^ we injected ID8-*Trp53*^-/-^/iRFP (KO) and ID8-*Trp53*^-/-^/*Igf2bp1*^-/-^/iRFP (DKO) cells intraperitoneally into both immunocompetent C57BL/6 and immunodeficient *Foxn1*^nu^ mice (Fig. 3a). Ascitic fluid formation served as the termination criterion.^36^ Each cell lines formed peritoneal lesions in both mouse strains, and tumor burden—assessed by ascites volume and peritoneal iRFP fluorescence intensity at termination—was comparable (Supplementary Fig. S3a). However, in C57BL/6, *Igf2bp1* deletion (DKO) delayed ascites formation and doubled the median survival time compared to KO cells (Fig. 3a and Supplementary Fig. S3a). In contrast, *Igf2bp1* loss only modestly reduced tumor growth in *Foxn1*^nu^ mice, likely reflecting the pro-proliferative role of *IGF2BP1* and the absence of immune-mediated pressure in this model.

Next, we isolated iRFP-positive DKO cells from ascitic fluid of C57Bl/6 mice and found significantly higher MHC-I/*PD-L1* ratios than in KO cells, which correlated with increased cytotoxic T cell numbers (Fig. 3b and Supplementary Fig. S3b). In addition, TCGA RNA-seq data revealed a negative correlation between *IGF2BP1* expression and intratumoral CTL infiltration in human HGSC, with *IGF2BP1* levels more strongly associated with CTL exhaustion than activation (Supplementary Fig. S3c, d). Multispectral imaging (MSI) of an independent HGSC-tissue microarray (TMA) further confirmed that higher *IGF2BP1* expression correlated with reduced CTL infiltration and greater T cell to tumor cell distance (Fig. 3c and Supplementary Fig. S3e). Collectively, these findings indicate that *IGF2BP1* promotes HGSC progression by enhancing proliferation—via MYC- and E2F-driven gene expression^37,40^ —and by disrupting MHC-I and *PD-L1* regulation, fostering an immunosuppressive microenvironment that enhances immune evasion (Fig. 3d).

### IGF2BP1 limits MHC-I synthesis by promoting IRF1 protein decay

**Fig. 4:**
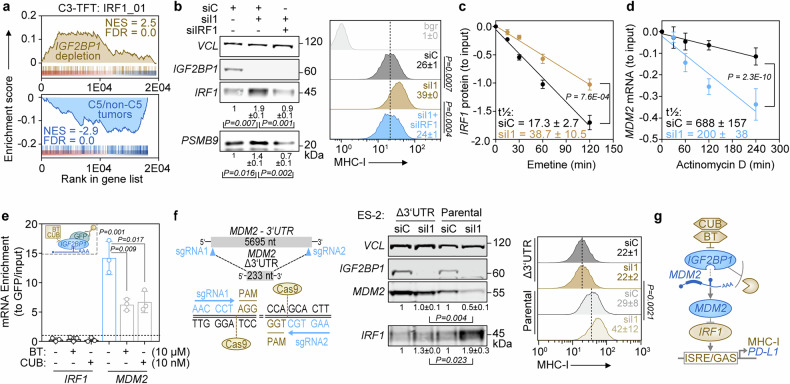
*IGF2BP1* limits *IRF1*-dependent transcription by preventing *MDM2* mRNA decay. **a** C5 tumors and *IGF2BP1*-depleted cells show inversed enrichment of *IRF1*-TFT gene sets. GSEA was performed upon log_2_ FC ranking of C5 to non-C5 tumors (blue) and *IGF2BP1* knockdown in ES-2 cells (yellow) using C3:TFT gene sets. **b** Phenotype rescue experiment of *IGF2BP1* depletion and co-depletion of *IRF1* in ES-2 cells analyzed by Western blotting (left) and flow cytometry (right) using indicated antibodies. Vinculin (VCL) served as loading control. **c** *IRF1* protein turnover was monitored in ES-2 cells by Western blotting upon *IGF2BP1* depletion and emetine treatment (100 µM) to block translation. **d** *MDM2* mRNA turnover was monitored in ES-2 cells by qRT-PCR upon *IGF2BP1* depletion and actinomycin D treatment (5 µM). **e** RNA-immunoprecipitation (RIP) using GFP antibodies in ES-2 with *IGF2BP1* deletion and re-expression of GFP-*IGF2BP1*. GFP served as negative control. Cells were treated with indicated compounds and concentrations or DMSO as negative control 24 h before the RIP experiment. Associated transcripts were quantified by qRT-PCR relative to inputs, controls and H2AC12. **f** The endogenous *MDM2*-3’UTR was deleted by CRISPR/Cas9 in ES-2 cells (left). Cells were analyzed by Western blotting (middle) and flow cytometry (right) upon *IGF2BP1* knockdown. **g** Schematic depicts *IGF2BP1*-directed control of *IRF1*-dependent transcription via *MDM2*. Error bars indicate s.d. for *N* ≥ 3 experiments. Statistical significance was assessed using Chi-squared comparison (**c**, **d**) or an unpaired two-tailed t test (**e**, **f**)

To investigate how *IGF2BP1* impairs MHC-I expression, we performed GSEA in C5 tumors and *IGF2BP1*-depleted ES-2 cells. We found an inverse correlation between immune-related and proliferative cancer hallmark gene sets (Supplementary Fig. S4a and Supplementary Table 7). Transcription factor target gene sets (TFT) indicated that C5 tumors exhibit an increased E2F-driven proliferative signature,^4^ whereas *IGF2BP1* depletion in ES-2 decreased E2F-driven transcription.^37^ In contrast, IRF-driven gene expression, particularly *IRF1*, was downregulated in C5 tumors but increased with *IGF2BP1* depletion (Fig. 4a, Supplementary Fig. S4b and Supplementary Table 8). Notably, *IGF2BP1* expression also negatively correlated with *IRF1* mRNA levels (Supplementary Fig. S4c) and IGF2BP1 depletion with the siRNA pool and an alternative siRNA increased IRF1 protein abundance (Supplementary Fig. S4d).

Given *IRF1*’s role in driving MHC-I and *PD-L1* transcription via *JAK*-*STAT*-mediated interferon signaling, we performed phenotype rescue experiments. *IGF2BP1* knockdown elevated *IRF1* protein levels, as well as the expression of the *IRF1*-driven immunoproteasome component *PSMB9* and MHC-I (Fig. 4b). Additional *IRF1* depletion restored *PSMB9* and MHC-I levels, suggesting *IGF2BP1* suppresses MHC-I presentation via *IRF1*. Surprisingly, *IGF2BP1* knockdown reduced *PD-L1* expression independently of *IRF1* (Supplementary Fig. S4e), consistent with findings in bladder and liver cancers.^23,41^

Although *IGF2BP1* typically regulates RNA turnover,^38^ *IRF1* mRNA stability remained unchanged upon *IGF2BP1* depletion (Supplementary Fig. S4f). Instead, *IRF1* protein half-life increased ~3-fold due to reduced protein decay (Fig. 4c and Supplementary Fig. S4g). *IRF1* protein turnover is regulated by *MDM2,*^42^ a known *TP53* antagonist (Supplementary Fig. S4h). We found that *IGF2BP1* and *MDM2* protein levels correlated in cytokeratin-positive tumor cells (Supplementary Fig. S4i). The knockdown of *IGF2BP1* reduced *MDM2* protein levels across ovarian cancer cell lines, regardless of *TP53* mutation status, increasing *IRF1* and *TP53* abundance (Supplementary Fig. S4j). Next, we measured the RNA stability and found that the *MDM2* mRNA half-life decreased ~4-fold following *IGF2BP1* depletion (Fig. 4d). *IGF2BP1*-CLIP data^38^ indicated specific *IGF2BP1* binding to the *MDM2* 3’UTR but not the *IRF1* mRNA. RNA co-immunoprecipitation (RIP) in ES-2 cells confirmed the binding of *IGF2BP1* to the *MDM2* mRNA (Fig. 4e and Supplementary Fig. S4k). This binding was disrupted by the small-molecule inhibitors BTYNB (BT) and Cucurbitacin B (CUB), which interfere with *IGF2BP1*-RNA interactions, reducing *MDM2* mRNA association with *IGF2BP1* (Fig. 4e).

The *MDM2* mRNA is a major miRNA target, and *IGF2BP1* promotes oncogene expression by antagonizing miRNA-directed degradation via the target mRNA’s 3’UTR.^38^ To test the 3’UTR dependency, we performed a CRISPR-mediated deletion of the *MDM2* 3’UTR. We found that this deletion increased *MDM2* protein levels but abolished the *IGF2BP1*-dependent regulation of *MDM2*, *IRF1*, and MHC-I, while *PD-L1* levels remained unaffected (Fig. 4f and Supplementary Fig. S4l). In line, *MDM2*-3’UTR luciferase reporter activity decreased with *IGF2BP1* knockdown or BT/CUB treatment (Supplementary Fig. S4m). These findings demonstrate that *IGF2BP1* promotes immune evasion by stabilizing *MDM2* mRNA in a miRNA- and 3’UTR-dependent manner, accelerating *IRF1* protein decay to suppress MHC-I presentation while sustaining *PD-L1* expression to uncouple CTL inhibitory from activating signals (Fig. 4g).

### IGF2BP1 promotes PD-L1 mRNA stability

**Fig. 5:**
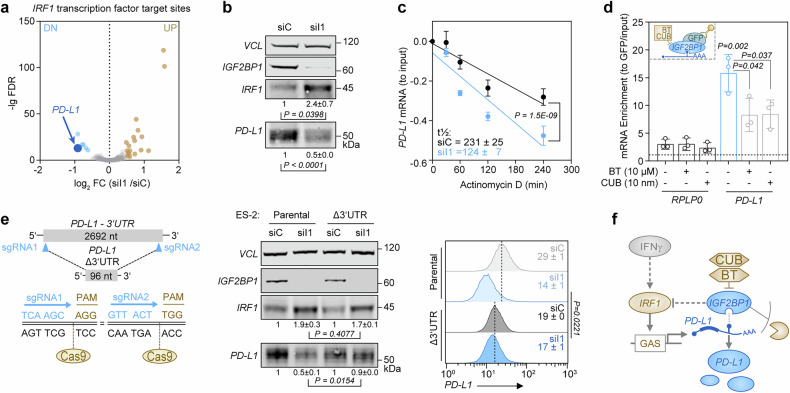
IGF2BP1 controls *PD-L1* mRNA turnover in a 3’UTR-dependent manner. **a** Volcano plot of *IRF1*-driven transcription (TFT: *IRF1*_01) upon *IGF2BP1* depletion in ES-2 cells. Transcripts with significant fold change (log_2_ FC > 2; FDR < 0.05) are labeled by color. **b** Western blot of *IGF2BP1* depleted ES-2 cells with indicated antibodies. Quantifications are shown below panels. **c** *PD-L1* mRNA turnover was monitored in ES-2 cells by qRT-PCR upon *IGF2BP1* depletion and actinomycin D treatment (5 µM). **d** RIP analyses using ES-2 cell extracts and treatments were performed as described in (Fig. 4e). **e** The endogenous *PD-L1*-3’UTR was deleted by CRISPR/Cas9 in ES-2 cells (left). Cells were analyzed by Western blotting (middle) and flow cytometry (right) upon *IGF2BP1* knockdown as indicated. Error bars indicate s.d. of *N* ≥ 3 experiments. Statistical significance was assessed using Chi-squared comparison (c) or an unpaired two-tailed t test (**b**, **d**, **e**). **f** Schematic depicts *IGF2BP1*-directed post-transcriptional control of *PD-L1* expression, uncoupling it from *IRF1*-dependent transcription

*PD-L1* is a known *IGF2BP1* target mRNA in liver and bladder cancer.^23,41^ In HGSC cells, the depletion of *IGF2BP1* consistently downregulated *PD-L1* mRNA expression while enhancing *IRF1*-driven gene signatures (Fig. 5a). Accordingly, *IGF2BP1* depletion reduced *PD-L1* protein abundance (Fig. 5b) and surface presentation (cf. Fig. 2c and Supplementary Fig. S2b for the alternative siRNA). To investigate the underlying mechanism, we measured *PD-L1* mRNA stability and found that *IGF2BP1* knockdown increased *PD-L1* mRNA decay (Fig. 5c). RIP assays confirmed *IGF2BP1*’s selective, druggable association with the *PD-L1* mRNA (Fig. 5d). Given that *PD-L1* expression is also regulated by miRNAs targeting its 3’UTR, we tested the 3’UTR dependency using luciferase reporter assays with the *PD-L1* 3’UTR and found reduced activity upon *IGF2BP1* depletion or treatment with *IGF2BP1* inhibitors (Supplementary Fig. S5a). miRNA-Trapping by RNA affinity purification (miTRAP) in ES-2 cell lysates confirmed *IGF2BP1* binding to the *PD-L1* 3’UTR and identified tumor-intrinsic miRNAs, including the miR17-92 family—known *PD-L1* regulators^43^—enriched in C5-HGSC (Supplementary Fig. S5b). To validate this finding, we performed genome editing to delete the *PD-L1* 3’UTR and found that this deletion abolished *IGF2BP1*-dependent regulation of *PD-L1* in parental *vs*. 3’UTR-deleted cells (Fig. 5e and Supplementary Fig. S5c).

Supporting *IGF2BP1*’s role in decoupling *IRF1*-driven *PD-L1* expression, *IRF1* and *PD-L1* mRNA levels strongly correlated in immune-responsive C2 tumors but showed a weaker correlation in immune-suppressed C5 tumors (Supplementary Fig. S5d). MSI analysis of an HGSC-TMA confirmed a significant correlation of *IGF2BP1* protein with *PD-L1* protein expression in tumor cells, but not in T cells (Supplementary Fig. S5e). These findings establish *IGF2BP1* as a key regulator of immune evasion in HGSC by disrupting *IRF1*-driven MHC-I synthesis while promoting *PD-L1* expression through a miRNA- and 3’UTR-dependent mechanism (Fig. 5f).

### IGF2BP1 inhibition synergizes with PD-1 blockade

**Fig. 6:**
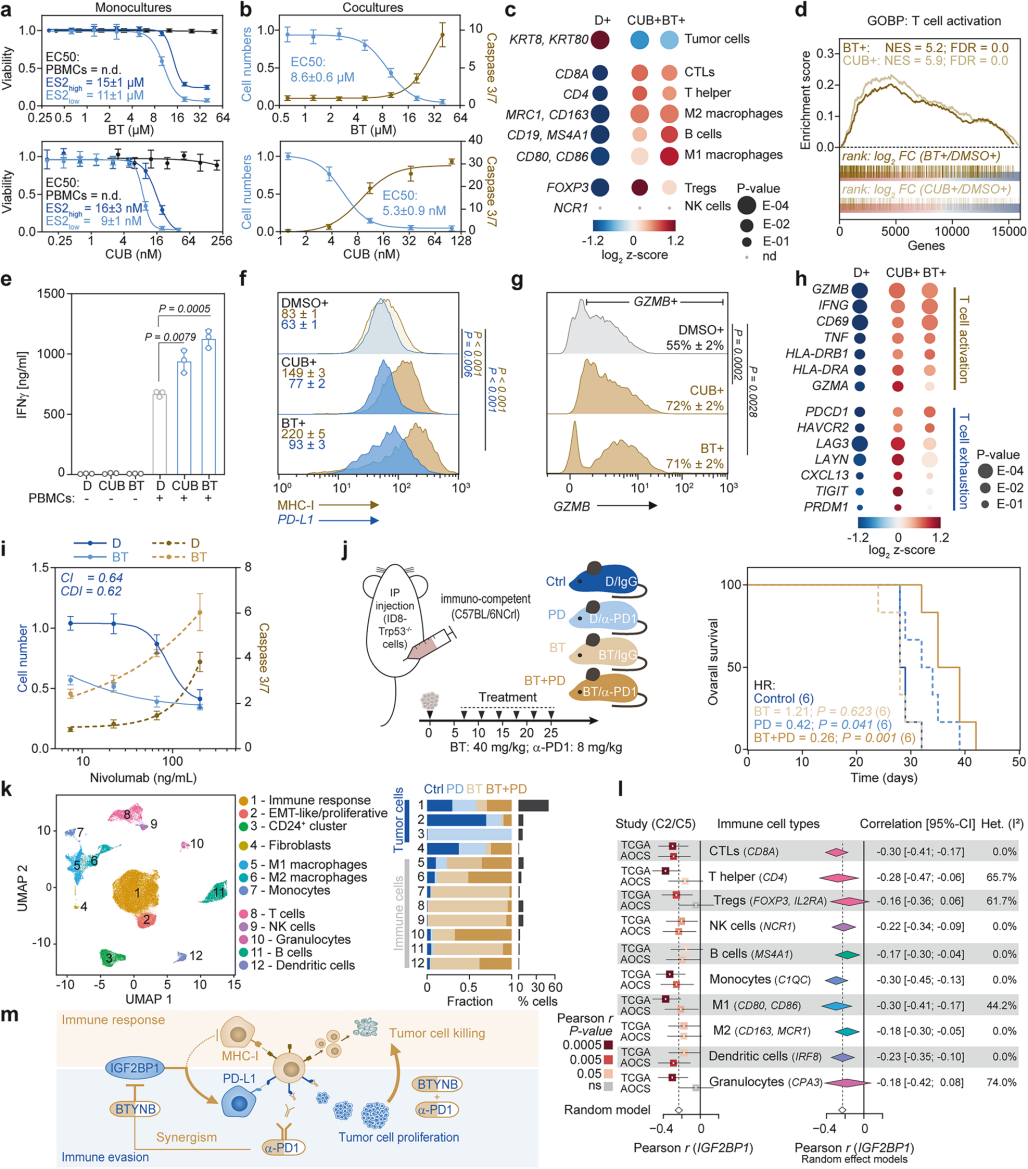
IGF2BP1 inhibition promote T cell-mediated tumor cell killing and synergizes with PD-1 blockade. **a** ES-2 cells (low density: 6.500 cells/cm²; or high density: 32.500 cells/cm²) or human PBMCs (32.500 cells/cm²) were treated with indicated concentrations of BT or CUB for 72 h before cell viability was determined by confluence measurements using the Incucyte S3, Cell Titer Glo or propidium iodide staining to determine EC_50_. Error bars indicate s.d. from *N* ≥ 3 experiments. **b** Cocultures of ES-2 cells with HLA-matched PBMCs were cultured for 36 h in the presence of caspase 3/7 green indicating T cell-mediated tumor cell killing. **c** Cocultures of ES-2 cells and PBMCs treated with BT (10 µM) or CUB (10 nM) for 36 h were analyzed by RNA-seq. Tumor and immune cell content is shown by indicated marker expression as a bubble chart. **d** GSEA of RNA-seq data from cocultures (c) revealed the enrichment of GOBP gene sets related to T cell activation (cf. Supplementary Table T9). **e–g** Cocultures of ES-2 cells and PBMCs were treated as in (**c**). ELISA (**e**) of cleared supernatants was used to determine IFNγ secretion of T cells. Flow cytometry with indicated antibodies (**f**) was used to assess alterations in MHC-I and *PD-L1* presentation on tumor cells. Intracellular *GZMB* staining (**g**) of fixed tumor cells analyzed by flow cytometry are shown as histograms. Numbers indicate MFI (f) or percentage of cells (**g**). S.d. and statistical significance by unpaired two-tailed t testing was determined for *N* ≥ 3 experiments. **h** Indicated T cell activation and exhaustion markers from RNA-seq experiments (**c**) are shown as a bubble chart. **i** Cocultures of ES-2 cells with HLA-matched PBMCs were treated with indicated concentrations of Nivolumab in the absence (0.05% DMSO) or presence of BT (5 µM). Relative tumor cell numbers and T cell-mediated tumor cell killing monitored by caspase 3/7 green was used to determine EC_50_ concentrations. Synergism was evaluated by the combination index (CI)^45^ and the coefficient of drug interaction (CDI).^46^ Synergy is indicated for values <1. **j** Schematic illustrates treatment groups and regimens of ID8/*Trp53*-/- cells engrafted in immuno-competent mice (left). Differences in the survival of each treatment group are shown as Kaplan–Meier analyses with HR values < 1 indicating survival benefits (right). Numbers of mice per condition are indicated. Statistical significance was determined by logrank testing. **k** Ascites fluids from 6 mice per treatment (**j**) were pooled and analyzed by scRNA-seq. UMAP plot shows identified cell clusters (left). Cluster distribution across treatment conditions (fraction; middle) and proportional cell content (% cells; right) are presented as a bar diagram. **l** Pearson correlations of *IGF2BP1* expression and indicated immune subsets were analyzed in C2 and C5 subtypes of the TCGA-RNAseq cohort (*n* = 121) and the AOCS dataset (*n* = 105) using indicated marker genes. Results are presented as a forest plot showing Pearson’s r with 95% confidence intervals; box sizes reflect sample size, and color indicates significance of the correlation (left). Random effect models were applied for each immune subset across datasets to estimate the overall correlation and heterogeneity indicated by *I²* (right). **m** Schematic illustrates the potential mechanism, how the *IGF2BP1*-directed inhibitor BT might synergize with PD-1 blockage to promote T cell-mediated killing of HGSC cells

The immune-evasive yet druggable nature of *IGF2BP1* in ovarian cancer cells suggests that inhibiting *IGF2BP1*-RNA interactions with small-molecule inhibitors such as BT or CUB may enhance T cell-mediated tumor cell killing. To test this, we determined the EC_50_ values of BT and CUB in monocultures of ES-2 and cocultures with human PBMCs. We found that in monocultures, both compounds inhibited ES-2 cell growth at nano- to micromolar concentrations (Fig. 6a), while PBMC viability remained unaffected even at 5- to 20-fold higher BT and CUB doses, consistent with their negligible *IGF2BP1* expression (Supplementary Fig. S6a).

In cocultures of ES-2 cells with HLA-matched PBMCs, we found that BT and CUB treatment reduced EC_50_ values compared to monoculture and increased caspase 3/7 activity in a dose-dependent manner (Fig. 6b), indicating enhanced T cell-mediated tumor cell killing. This trend was consistent across C5-like cell lines (OVCAR-8 and TOV112D) and even non-C5-like COV318 cells (Supplementary Fig. S6b). *IGF2BP1* inhibition was associated with a higher T cell-to-tumor cell ratio, as confirmed by flow cytometry and RNA-seq of FACS-separated tumor and immune cells (Fig. 6c and Supplementary Fig. S6c). To further characterize immune cell dynamics, we analyzed cell populations upon BT or CUB treatment by RNA-seq and found that keratin-positive tumor cells decreased, while immune cell populations, particularly CTLs and T helper cells, increased (Fig. 6c). Both compounds enhanced CTLs and T helper cells. Moreover, BT also increased M1 macrophages and B cells, known to support immune responses.^8^ In contrast, CUB strongly elevated Tregs, suggested to be immune suppressive in HGSC.^44^ NK cells (NCR1⁺) were absent in these cocultures.

To assess T cell activation, we performed GSEA and observed enhancement of immune-related Hallmark gene sets, as well as Gene Ontology biological processes (GOBP) with T cell activation and response (Supplementary Fig. S6d and Supplementary Table 9). Enhanced T cell activation of BT- and CUB-treated cocultures was confirmed by elevated IFNγ secretion, determined by ELISA (Fig. 6e). This IFNγ upregulation increased MHC-I on tumor cells with minimal *PD-L1* induction, resulting in an immune-responsive MHC-I/*PD-L1* ratio (Fig. 6f). We also found that caspase 3/7 activity correlated with elevated *GZMB* levels in tumor cells (Fig. 6g), supporting enhanced T cell-mediated killing. However, while both compounds promoted T cell activation, exhaustion markers—except for PD-1 (*PDCD1*) and *HAVCR2*—were more prominent in CUB-treated cocultures (Fig. 6h). These findings demonstrate that *IGF2BP1* inhibition disrupts immune evasion by activating T cells and enhancing tumor cell killing. Together, these results highlight the potential of RBP-targeted therapies in reactivating anti-tumor immunity in ovarian cancer cells in vitro.

To test if *IGF2BP1* inhibition could enhance therapeutic efficacy of PD-1 blockade, we treated cocultures with BT and Nivolumab. We found that monotherapies had EC_50_ values of 8.6 µM for BT and 87.7 ng/mL for Nivolumab (Fig. 6a, i), while the combination treatment of 5 µM BT reduced Nivolumab’s EC_50_ value by ~17-fold to 5.3 ng/mL. To evaluate potential synergy, we applied the combination index (CI) ^45^ and coefficient of drug interaction (CDI) methods.^46^ CI analysis revealed a 36% greater inhibition than expected (CI = 0.64), while CDI analysis showed a 38% stronger effect (CDI = 0.62), confirming synergy between the two inhibitors (Fig. 6i).

To evaluate *IGF2BP1* inhibition and BT/anti-PD-1 synergy in vivo, we used the syngeneic ID8/*Trp53*^-/-^ mouse model. Mice received monotherapies or combination therapy starting seven days post-engraftment (Fig. 6j). We found that the combination treatment reduced the hazard ratio by ~40% compared to anti-PD-1 (clone RMP1-14) monotherapy (Fig. 6j). Likewise, median survival improved by ~30% compared to the control group and by 15% compared to anti-PD-1 monotherapy. Although BT was active in vitro, it did not significantly improve survival as a monotherapy in vivo, likely due to rapid ascites development necessitating early termination. However, ascitic fluid composition varied substantially between treatment groups.

To dissect treatment-dependent changes in cell composition and gene expression in vivo, we performed scRNA-seq analyses on ascitic fluids and identified distinct tumor and immune cell clusters, which substantially varied between treatment conditions (Fig. 6k and Supplementary Fig. S7a). Control and anti-PD-1-treated mice showed tumor cell dominance (>90%), whereas BT and combination therapy groups contained only 28% and 67% tumor cells, respectively. Based on marker gene expression (cf. Fig. 1d), we classified tumor cells into immune-responsive, EMT-like/proliferative, and Cd24⁺ clusters (Supplementary Fig. S7b). We found that tumor cells from BT- or combination-treated mice were predominantly immune-responsive, whereas those from control animals exhibited a proliferative signature (Fig. 6k and Supplementary Fig. S7a, b). Notably, anti-PD-1 monotherapy favored Cd24⁺ cells (Fig. 6k), which underwent a metabolic shift from oxidative phosphorylation to glycolysis (Supplementary Fig. S7a, b and Supplementary Table 10), highlighting their metabolic adaptability.^19^ Gene expression analysis showed enhanced immune receptor signaling in tumor cells from BT- or combination-treated mice, whereas control and anti-PD-1 monotherapy groups were enriched in RNA metabolism, translation, and cell cycle-related pathways (Supplementary Fig. S7b).

Consistent with reduced tumor cell content, BT and combination groups displayed increased immune cell accumulation in the ascitic fluid, with BT alone eliciting the highest enrichment (>70%; Supplementary Fig. S7c). This heightened immune activity may contribute to the more rapid ascites formation that limits survival benefit. To further investigate treatment-specific effects on immune subsets, we focused on T and NK cell clusters (Supplementary Fig. S7d). NK cells represent a small cluster, predominantly detected and active in BT and combination treatment groups (Supplementary Fig. S7e). The T cell cluster—comprising CTLs, T helper, Tregs, and other T cell subsets (Supplementary Fig. S7d)—was mainly present in BT and combination groups as well (Supplementary Fig. S7f). Control and anti-PD-1 cohorts contained <1% Cd45⁺ lymphocytes and no *Cd8a*⁺ CTLs were detected in ascitic fluid following anti-PD-1 treatment alone (Supplementary Fig. S7f). Although total T cell abundance was lower in the combination group compared to BT alone, the proportion of CTLs was highest among all treatments. Furthermore, T cells from the combination treatment were more activated yet also displayed higher exhaustion levels (Supplementary Fig. S7f).

To validate *IGF2BP1*’s association with an immune-suppressive tumor microenvironment in human HGSC, we correlated *IGF2BP1* mRNA expression with immune subsets identified in our mouse scRNA-seq dataset using the C2 and C5 sub-types from two public datasets (Fig. 6l). *IGF2BP1* expression showed a broadly negative correlation with immune cell abundance, most consistently and significantly for CTLs and monocytes (Fig. 6l).

Collectively, these findings provide the first evidence that *IGF2BP1* inhibition enhances immune checkpoint therapy by disrupting *IGF2BP1*-driven uncoupling of *IRF1*-dependent MHC-I/*PD-L1* synthesis in tumor cells (Fig. 6m). Thus, BT treatment is beneficial for checkpoint therapies such as PD-1 blockade both in vitro and in vivo.

## Discussion

ICTs have significantly improved outcomes in cancers like melanoma and lung cancer. However, their efficacy remains limited in HGSC, highlighting the urgent need to uncover mechanisms of immune evasion. In immune-desert HGSCs, reactivating antitumor immunity and identifying ICT-supportive strategies are pressing goals.^7,8^ Typically, tumor immune escape results from an immunologically cold microenvironment and tumor-intrinsic factors, including an unfavorable MHC-I/PD-L1 ratio.^47^

Our study identifies, for the first time, oncofetal and stemness-promoting RBPs as central to the immune-desert C5-HGSC subtype. In addition to a skewed MHC-I/*PD-L1* balance, this subtype exhibits pronounced T cell exhaustion amid sparse immune infiltrates. Identified oncofetal RBPs were most prevalent in EMT-like, proliferative, neuronal-like, *CD24*^+^, and invasive tumor cell clusters. Notably, *CD24*^+^ stem cells with high metabolic plasticity and the neural circuit have recently been linked to immune evasion in ovarian cancer.^19,20^ Oncogenic RBPs are key posttranscriptional regulators that function as oncogenic protein nodes in various cancers.^15^ This nodal function relies on RBP-coordinated cross-talk among multiple tumor cell–intrinsic signaling pathways,^18,26,30,38,48,49^ including those linked to immune-related signaling.^25,27,28,41^ Thus, some oncogenic RBPs, including those identified here, exemplify the “by-the-numbers” principle of cancer aggressiveness^15^—a concept particularly relevant to C-class tumors.^4^ Notably, the vast majority of HGSC cases are classified as C-class malignancies. Given their unique expression patterns^22^ and emerging druggability,^16^ RBPs represent promising targets for therapy-resistant and immune-desert ovarian cancers. The C5-HGSC subtype is defined by upregulation of at least eight RBPs—including IGF2BPs, LIN28s, and MSIs—that drive stemness and immune evasion.^22,24,26^ This RBP signature distinguishes C5 from non-C5 tumors as accurately as the established subtype classification.^9^ At the molecular level, the identified oncogenic RBPs contribute to immune escape to varying extents. An siRNA screen confirmed that these RBPs promote an unfavorable MHC-I/*PD-L1* ratio, reinforcing tumor immune resistance.^47^

Among them, *IGF2BP1* emerged as a dominant immune evasion driver in C5-like ovarian cancer. We demonstrate a novel mechanism where *IGF2BP1* post-transcriptionally uncouples *IRF1*-driven MHC-I and *PD-L1* expression. By stabilizing *PD-L1* mRNA while promoting *MDM2*-dependent *IRF1* degradation, *IGF2BP1* interferes with IFN signaling in HGSC. Given that *MDM2* and *PD-L1* mRNAs are major miRNA targets and regulated via m^6^A RNA modifications,^41,50^ and that IGF2BP1 is a well-known m^6^A reader, our findings emphasize the role of post-transcriptional mechanisms in immune evasion. Although *IGF2BP1*’s gene regulation is not strictly m^6^A-dependent,^18^ m^6^A supports its RNA-stabilizing activity for key oncogenes.^40,49^ Critically, the data support a model in which IGF2BP1 acts as a central integrator of oncogenic signaling and immune suppression. Beyond regulating PD-L1, IGF2BP1 likely modulates a broader network of immune checkpoints and cytokine pathways, as suggested by its RNA interactome. The convergence of m^6^A modification, RBP activity, and immune signaling defines a previously underappreciated regulatory axis in HGSC.^38,40,49^

Pharmacological disruption of *IGF2BP1*-RNA interactions with small molecules such as BT and CUB abolishes its binding to *MDM2* and *PD-L1* mRNAs, mirroring effects seen with *E2F1-3* and *MYCN.*^39,51^ These inhibitors impair tumor cell viability while sparing T cells and restore antitumor immunity by enhancing CTL and T helper cell activation. Notably, both compounds show minimal cytotoxicity toward immune cells, which do not express IGF2BP1, highlighting their suitability as adjuncts to enhance checkpoint therapy efficacy. While BT also promotes infiltration of B cells and M1 macrophages, CUB—previously reported to exert both pro- and anti-inflammatory effects^52^—increases Tregs, which may counterbalance therapeutic efficacy.^8^

These findings are consistent with experimental liver cancer models^23^ and highlight the relevance of the tumor cell intrinsic regulation of MHC-I/*PD-L1* in ovarian cancer.^47,53^

Crucially, this study provides the first evidence that *IGF2BP1* inhibition by BT synergizes with ICT, particularly PD-1 blockade via Nivolumab in vitro and in vivo. This mirrors findings in syngeneic melanoma models where *Igf2bp1* knockdown enhanced ICT responsiveness.^27^ scRNA-seq analysis revealed that BT boosts immune infiltration and reactivation beyond that achieved by PD-1 blockade alone. Though BT monotherapy was well tolerated, it did not extend survival, likely due to inflammatory signals accelerating ascites formation. However, combining BT with PD-1 inhibitors significantly improved survival and prevented the outgrowth of *CD24*⁺ tumor cells, which are resistant to conventional therapies and escape immune clearance via *SIGLEC-10*-mediated macrophage suppression.^19,54^ These results emphasize the potential of combining RBP inhibition with ICT to reprogram immune-desert tumors. Mechanistically, this approach may enhance antigen presentation, restore interferon signaling, and reshape the TME toward a more inflamed state. However, the therapeutic window for RBP inhibitors requires careful optimization to avoid unwanted systemic effects. The development of next-generation, context-selective RBP inhibitors or Proteolysis Targeting Chimeras (PROTACs) could provide improved specificity and safety.^16,55^

With continued research^56^ and development of RBP-targeting agents and PROTAC strategies,^16,55^ immunotherapy could become a viable option for a broader spectrum of ovarian cancer patients—especially those with immune-desert phenotypes currently unresponsive to ICT. While clinical translation requires further validation, our findings reveal a promising therapeutic avenue in targeting RBPs to restore anti-tumor immunity in immunologically cold HGSC. This strategy not only enhances MHC-I/*PD-L1* regulation, promotes immune infiltration, and sensitizes tumors to ICT, but may also be applicable to other malignancies with RBP-driven immune suppression, such as endometrioid cancer and PDAC. Although BT remains an experimental compound, CUB is a natural product already approved by the Chinese Food and Drug Administration,^52^ and additional agents aimed at RBP pathways—such as METTL3 inhibitors already advancing into clinical trials (NCT05584111)^57^—may likewise help reinvigorate anti-tumor immunity. Thus, the concept of RBP inhibition as a means to potentiate ICT without compromising immune functionality represents a translatable approach, opening the door to next-generation combination therapies beyond ovarian cancer.

## Materials and methods

### Human studies

This paper analyzes publicly available RNA sequencing (RNA-seq) data from The Cancer Genome Atlas (TCGA), original research articles describing tumor datasets (GSE9899, GSE147980, GSE204748) and cell experiments (GSE109605, GSE146807). Human HGSC samples, collected from patients undergoing surgery, were granted after written informed patient consent and ethical approval by the Ethics Committee of the Medical Faculty/Martin Luther University Halle/Wittenberg (208/17.06.09/12). This study generated RNA-seq (GSE234708) and scRNA-seq datasets (GSE285177). Multi-spectral imaging was conducted using commercially available TMA. Available patient information is summarized in Supplementary Table 1.

### Mouse models and animal handling

Animals were handled according to the Martin Luther University guidelines. Permission was granted by the local ethical review committee (reference numbers: 42502-2-1381 MLUs; 42502-2-1574 MLU). Immunocompetent (C57BL/6NCrl) or immunodeficient athymic nude mice (FOXN1nu*/*nu) were obtained from Charles River. ID8*/Trp53* knockout (KO)^36^ or ID8*/Trp53/Igf2bp1* double knockout (DKO) cell lines stably transduced with iRFP encoding lentiviruses were harvested in PBS and 5 × 10^6^ viable cells were IP-injected into six-week-old female mice. Where indicated, mice received IP injections starting 7 days after tumor cell implantation, twice a week, as follows: BT (40 mg/kg body weight in 100 µL PBS solution containing 30% cyclodextrin and 10% DMSO) and/or anti-PD-1 (8 mg/kg antibodies (clone RMP1-14) in 100 µL PBS). DMSO and/or IgG2a antibodies served as controls. All mice were examined daily and euthanized when palpable ascites reached the endpoint criterion of approximately 3 mL. Ascitic fluid was collected, and its volume was measured. IP tumor burden was monitored via near-infrared imaging (Pearl Trilogy Imaging System, LI-COR) and quantified using ImageStudio Lite software (LI-COR). Ascitic fluid from immunocompetent mice was depleted of erythrocytes using Red Blood Cell Lysis Solution (Miltenyi Biotec) before cells were cryopreserved in MACS Freezing Solution. Flow cytometry analyses were performed using a MACSQuant Analyzer with *Cd3/Cd8* antibodies to assess T cell content as described below. Immune receptor presentation on iRFP-positive tumor cells was analyzed using *H2* (MHC-I) and *PD-L1* antibody staining as described below. scRNA-seq was performed using the BD Rhapsody HT as described below.

### Molecular subtyping

Five independent HGSC tumor cohorts (TCGA-OV;^4^ Local (GSE147980);^48^ Sood (GSE204748),^17^ TCGA-OV-Agilent (additional cases);^4^ and AOCS (GSE9899)^9^) were obtained from R2, and assigned to subtypes (C1 (mesenchymal), C2 (immune reactive), C4 (differentiated), and C5 (proliferative/low-immune-response)) based on gene expression profiles. Samples assigned to more than one cluster (cut-off: delta-NES < 0.4) and non-HGSC samples were excluded from subsequent analyses.

To determine C5-specific features GSEA was performed using indicated MsigDB gene set collections. For each subtype, changes in CNV and mutational counts were analyzed using data derived from cBioPortal. Average microRNA abundances are summarized for the individual microRNAs or microRNA seed families. Where indicated, z-score transformation was applied to analyze gene expression.

### RNA isolation, RNA sequencing and gene expression analyses

Total RNA was isolated from cell experiments using TRIzol®, as previously described.^58^ RNA integrity was assessed using a Bioanalyzer 2100 (Agilent). RNA-seq libraries were prepared according to the manufacturer’s instructions (Illumina or Lexogen). For mRNA-seq, total RNA was used as input for poly(A) enrichment with oligo(dT) beads. Library preparation and paired-end sequencing were performed by Novogene (Hong Kong) on the Illumina HiSeq or NovaSeq platforms, or by the Core Facility Imaging (MLU Halle) on the Illumina NextSeq 1000 platform. For miTRAP analyses, single-end sequencing was performed on Illumina HiSeq 1500 platform at Novogene (Hong Kong).

Low-quality read ends and residual sequencing adapter fragments were trimmed using Cutadapt. Reads were aligned to the human genome (UCSC GRCh37 or 38) using TopHat2, HISAT2 or Bowtie2, respectively. Gene-mapped reads were summarized using FeatureCounts based on Ensembl (GRCh37.75 or GRCH38.89) or miRbase (V22). Further details are available associated to the respective datasets. Differential gene expression was determined by DESeq2 using Galaxy^59^ for mRNA sequencing, or EdgeR^60^ for small RNA sequencing. GSEA was performed on pre-ranked lists using protein-coding genes.^61^

### scRNA sequencing

HGSC specimens were dissociated with the Tumor Dissociation Kit (Miltenyi Biotec) using the soft tissue protocol, filtered (30 µm) and erythrocyte-depleted using the Red Blood Cell Lysis Solution (Miltenyi Biotec) before cryopreservation with MACS Freezing Solution (Miltenyi Biotec) until further processing. Cells from murine ascites fluids were collected by centrifugation (500 *g*; 5 min). Dead cells were removed using the Dead Cell Removal Kit (Miltenyi Biotec), before single-cell capture using the BD Rhapsody HT system (BD Biosciences). Cell viability, monitored by calcein and Draq7 staining, ranged between 90 and 95% for captured single cells. cDNA synthesis and whole transcriptome library construction was performed according to the manufacturer’s instructions (BD Biosciences). Paired-end sequencing was conducted on an Illumina NextSeq 1000 platform. FASTQ files were processed via Seven Bridges using the BD Rhapsody Sequence Analysis Pipeline v2.0. Putative multiplet cells were identified using the R-package scDblFinder.^62^ Quality filtering, normalization, and cell clustering was performed using the R-package Seurat.^63^ Cells expressing fewer than 200 distinct genes or more than 33% mitochondrial gene content, as defined by MitoCarta v3,^64^ were removed from further analyses. Expression values were normalized using Seurat’s SCTransform v2 algorithm. An integrated data set, normalized expression values, generation of UMAPs and marker gene expression was conducted via the Seurat R-package. Cluster marker genes with a log_2_ fold-change expression > 0.5 are shown in Supplementary Table 3. GSEA was performed using marker gene expression and log_2_ fold-changes in gene expression between individual cell clusters to determine cluster-specific cell properties. NES scores are shown in Supplementary Table 4 for human HGSC samples and Supplementary Table 10 for the mouse model.

### Cell line classification

RNA-seq data for human ovarian cancer-derived cell lines were obtained from the CCLE via R2. Hypermutated cell lines,^32^ and cell lines with wild-type or unknown TP53 status were excluded. For the 28 remaining cell lines, GSEA was performed based on log_2_ fold-changes in gene expression relative to the cohort median to determine the properties of C5-related genes. Cell line-ranking based on NES scores was conducted with indicated gene sets. From the top-ranking cell lines, OVCAR-8, ES-2 and endometrial ovarian cancer-derived TOV112D were selected for further experiments. COV318 was chosen as immune-responsive HGSC cell line with high RBP expression.

### Human and murine cell lines

All cells were cultured at 37 °C with 5% CO₂. All human ovarian cancer-derived cell lines (ES-2 (CVCL_3509), TOV112D (CVCL_3612), OVCAR-8 (CVCL_1629), and COV318 (CVCL_2419)), and their derivatives were maintained in DMEM supplemented with 10% FBS and 1% GlutaMAX. Human PBMCs, as well as PBMC-tumor cell cocultures, were cultured in RPMI supplemented with 10% FBS, 1% GlutaMAX, 1% penicillin/streptomycin, and 10 nM IL-2. For lentivirus production, HEK293T cells (CVCL_0063) were cultured in Opti-MEM supplemented with 2.5% FBS, 1% GlutaMAX, and 1% sodium pyruvate. Murine cell lines (ID8 (CVCL_IU14)) and derivatives were maintained in DMEM supplemented with 4% FBS, 1% GlutaMAX, 1% Insulin-Transferrin-Selenium, and 1% penicillin/streptomycin.

### Transfections and treatments

Cell transfections with DNA or siRNAs were performed using Lipofectamine 3000 or Lipofectamine RNAiMAX (Thermo Fisher Scientific) according to the manufacturer’s instructions and previously described.^38^ For the siRNA screen, pools of 5 siRNAs per RBP were used. For further *IGF2BP1* depletions, a pool of 12 siRNAs (I1) or an alternative single siRNA directed against the *IGF2BP1*-3’UTR (3p), which is not included in the pool, was used. All plasmids, oligonucleotides and siRNAs used in this study are summarized in Supplementary Table 11. For genomic deletions via CRISPR/Cas9, from cells, co-transfected with GFP-T2A-Cas9 and two RFP-sgRNA-encoding plasmids, GFP/RFP-positive single-cell clones were generated by FACS using a FACSMelody (BD Biosciences). *IGF2BP1*-KOs in ES-2 cells^18,38^ and Trp53-KO in ID8 cells^36^ were previously described.

For lentiviral particle production, 3.5 × 10^6^ HEK293T cells were transfected using Lipofectamine 3000 with the packaging plasmids psPax2, pMD2.G, and the pLVX lentiviral expression vector encoding iRFP, GFP, or GFP-*IGF2BP1*. Virus titers were determined 48 h post-infection by flow cytometry using a MACSQuantX Analyzer (Miltenyi Biotec). Transduction was performed at 10 MOI.

BT was synthesized as previously described.^39,65^ Purity of the synthesis product in shown by HPLC in Supplementary Fig. S8. BT, CUB (Selleckchem), and the PD-1-blocking antibody Nivolumab (DC Chemicals) were used at the indicated concentrations and incubation times. RNA decay analysis using 5 µM actinomycin D (Sigma-Aldrich) or protein turnover experiments using 100 µM emetine (Sigma-Aldrich) were performed for the indicated time points 72 h post-siRNA transfection.

### Plasmids and cloning

All plasmids used and generated in this study are indicated in Supplementary Table 11. The 3’UTRs of *MDM2* and *PD-L1* were amplified by PCR using genomic DNA from ES-2 cells and indicated oligonucleotides containing stated restrictions sites for sub-cloning as previously described.^38^ Cloning strategies including plasmids, oligonucleotides and restriction sites are summarized in Supplementary Table 11.

### Coculture experiments, T cell killing assays and synergy analyses

For coculture experiments, target cells were cultured in RPMI medium supplemented with 10% FBS, 1% GlutaMAX and 1% penicillin/streptomycin. Cells were transfected or treated 48 h prior to the addition of human HLA-matched PBMCs from healthy donors (CTL Europe) at an immune-cell-to-target-cell ratio of 5:1. PBMCs were added along with RPMI activation medium containing 10 nM IL-2 and human T-Activator *CD3/CD8* Dynabeads™ (Thermo Fisher). Alternatively, pre-coated plates with Ultra-LEAF™-*CD3* and -*CD8* antibodies (BioLegend) were used for T cell activation. T cell-mediated tumor cell killing was determined in an Incucyte S3 (Sartorius) using caspase-3/7 green (Sartorius). Additionally, cells were subjected to flow cytometry, RNA-seq analyses, FACS, or ELISA (BioLegend) 36 hours after PBMC-addition.

To evaluate the impact of BT co-treatment on PD-1 inhibition by Nivolumab, two synergy models were established in the ES-2/PBMC coculture system. The EC_50_ concentrations for each mono- and combination treatment were determined using 5 µM BT with increasing concentrations of Nivolumab. The Combination Index (CI)^45^ was calculated as follows:

$${CI}=\frac{a}{A}+\frac{b}{B}$$

where A and B represent the EC_50_ values of the single treatments, while a and b correspond to the EC_50_ values of the respective compounds in the combination treatment. The coefficient of drug interaction (CDI)^46^ was calculated using the formula:

$${CDI}=\frac{{AB}}{A\,\times \,B}$$

where A and B represent the viability of single treatments, while AB represents the viability of the combination treatment at the same concentrations. For both models, synergy is indicated by values < 1.

### Flow cytometry

Immune receptor presentation on human and murine tumor cells was analyzed using flow cytometry. Tumor cells were transfected or treated 72 h before staining, while cocultures were performed for 36 h. Mouse ascites was depleted of erythrocytes before antibody staining. Tumor cells were harvested using Trypsin/EDTA. 2 × 10^5^ cells were stained in FACS buffer (1% BSA w/v in PBS) containing the indicated antibodies for 15 min at room temperature (RT). For coculture-derived cells, staining was performed for 1 h at 4 °C. Cells were washed twice in FACS buffer before flow cytometry analysis was performed using a MACSQuant Analyzer to determine mean fluorescence intensities (MFI) of viable, single cells. Tumor cells in ascites were identified using iRFP fluorescence. PBMCs were stained for *CD3* and *CD8* to determine CTL content. To analyze the content of tumor cell internal *GZMB*, cells were fixed with 4% PFA for 10 min at RT, before permeabilization with 0.5% TRITON X-100 for 3 min at RT and blocking in FACS buffer for 15 min. *GZMB* staining was performed for 30 min at RT and washed twice with PBS. The percentage of *GZMB+* cells was analyzed by flow cytometry. A summary of all antibodies used is provided in Supplementary Table 11. Gating strategies are provided in Supplementary Fig. S9.

### Western blotting

Changes in protein abundances were determined by infrared Western blotting analyses. Total protein from harvested cells was extracted using lysis-buffer (50 mM Tris–HCl (pH 7.4), 50 mM NaCl, 2 mM MgCl_2_, 1% SDS, 125 U/mL Benzonase). Protein concentration was determined, and equal amounts of total protein were size-separated on NuPAGE 4–12% Bis-Tris mini gels (Thermo Fisher) before being transferred onto a nitrocellulose membrane (Amersham) for Western blotting. Protein expression was analyzed using indicated primary antibodies with fluorescence-coupled secondary antibodies, followed by detection via infra-red scanning (Odyssey CLx, LICOR). Vinculin (*VCL*) served as loading control for normalization. All antibodies used are summarized in Supplementary Table 11. Uncropped western blot scans are provided in Supplementary Fig. S10.

### Multispectral imaging

Marker expression and immune cell infiltration in HGSC patient samples were analyzed using TMAs (for sample information cf. Supplementary Table 1). Slides were incubated at 65 °C for 3 h before de-waxing with xylol and ethanol. After washing, slides were fixed in 4% PFA for 20 min, followed by antibody heat retrieval using HIER6 or HIER9 at 96 °C for 20 min in a water bath. Serial antibody stainings were performed using the Opal7 system according to the manufacturer’s instructions. All antibodies used are summarized in Supplementary Table 11. Images were acquired with a Leica TCS-SP8X confocal microscope equipped with a white light laser and hybrid detectors at 40× magnification. Time gating was applied to minimize background fluorescence and cross-talk. Nucleus and cell segmentation, as well as tissue classification for pan-CK-positive tumor cells, pan-CK-negative stroma cells, and *CD3/CD8* double-positive CTLs, were conducted using QuPath^66^ (V4.3.0) to determine compartment-specific mean fluorescence intensities and spatial relationships between tumor, stroma, and immune cells.

### RNA Co-Immunoprecipitation (RIP) and RT-q-PCR

RIPstudies and qRT-PCR were essentially conducted as described before.^37,58^ Primer sequences are summarized in Supplementary Table 11. Relative RNA abundance was determined by the ΔΔCt method using RPLP0, GAPDH and HIST2H3A for normalization.

### miTRAP

MiTRAP experiments using 3´UTR of *PD-L1* or MS2 control RNA were essentially performed as described recently.^58^ In vitro transcription of RNA baits was performed on linearized DNA templates. miTRAP analyses 40 pmol of the *PD-L1* and 2.5 pmol MS2-control RNA were immobilized on amylose resin pre-coupled to MS2BP-MBP for 30 min at 4 °C. Cell-free extracts from 5 × 10^6^ ES-2 cells per condition generated in binding buffer (20 mM Tris, pH 7.5, 150 mM NaCl, 1.5 mM MgCl_2_, 8.6% glycerol and 0.05% NP-40) supplemented with protease inhibitor cocktail (1:200; Sigma Aldrich) were cleared by centrifugation and incubated with bait-coupled resin and supplements (11 mg/mL heparin, 1 mM DTT and 400 U/mL RNAsin (Promega)) for 30 min at RT. After three washes with heparin-supplemented binding buffer, protein–RNA complexes were eluted twice in 150 μl binding buffer supplemented with 15 mM maltose. For protein analysis, amylose resins were incubated with 25 μl of SDS-sample buffer supplemented with 10% DTT. For RNA applications a TRIzol® extraction was performed. Purified RNA was sent for short-read RNA sequencing to identify associated miRNAs as described above.

### Kaplan–Meier, ROC analyses and Pearson correlation

Changes in overall survival probabilities were determined via Kaplan–Meier analyses using indicated gene signatures for RBPs (*DDX25, ELAVL3, IGF2BP1, IGF2BP3, LIN28B, MEX3A, MKRN3, MSI1*), immune surface receptors (*HLA-A, HLA-B, HLA-C*, inverted *PD-L1*) or immune cell types. Analyses were conducted via KMplot using the ovarian cancer dataset with automated cutoff selection to determine Hazardous ratios (HR) by log-rank testing. Relapse-free survival probabilities for immune cell subsets were determined by GEPIA2021 using CIBERSORT deconvolution and quartile cutoffs. ROC analyses were used to distinguish C5 from non-C5 tumors with the help of the pROC package in Rstudio. The average expression for each gene of the C5- or RBP signatures was determined based on z-transformed expression values. Pearson correlation between indicated genes was used to determine co-occurrences. Significance was determined by a parametric two-sided T-test. Forest plots were generated using the meta package in Rstudio based on Pearson *r*. Heterogeneity between datasets is indicated by *I²*.

### Statistics

All experiments were performed at least in biological triplicates. Statistical significance was determined by an unpaired two-tailed t test on normally distributed data. Otherwise, a non-parametric Mann–Whitney-test was performed. For Kaplan–Meier analyses, statistical significance was determined by log-rank tests. One- or two-way ANOVA testing was used for group-wise comparison. Chi square comparison was used to test for statistical significance between arbitrary curves.

## Supplementary information

Supplementary Material

Supplementary tables

## Data Availability

This paper analyzes existing, publicly available RNA sequencing and microarray data from HGSC patients from the following data sets: The Cancer Genome Atlas (TCGA), AOCS (GSE9899), Sood (GSE204748) and Local (GSE147980). Original research articles were deposited in NCBI’s Gene Expression Omnibus SuperSeries (https://www.ncbi.nlm.nih.gov/geo/) under the following accession numbers: GSE109605 and GSE147980. The RNA- (GSE234708) and scRNA-seq datasets (GSE285177) were generated by this study. All relevant data are available within the paper and its Supplementary Information files or are available from the corresponding authors upon reasonable request. Gating strategies of flow cytometry analyses and uncropped original Western blot images are provided as last Supplementary Figures.

## References

[1] Bowtell, D. D. et al. Rethinking ovarian cancer II: reducing mortality from high-grade serous ovarian cancer. *Nat. Rev. Cancer* **15**, 668–679 (2015). 26493647 10.1038/nrc4019 PMC4892184

[2] Labidi-Galy, S. I. et al. High grade serous ovarian carcinomas originate in the fallopian tube. *Nat. Commun.* **8**, 1093 (2017). 29061967 10.1038/s41467-017-00962-1 PMC5653668

[3] Ahmed, A. A. et al. Driver mutations in TP53 are ubiquitous in high grade serous carcinoma of the ovary. *J. Pathol.* **221**, 49–56 (2010). 20229506 10.1002/path.2696 PMC3262968

[4] Cancer Genome Atlas Research Network. Integrated genomic analyses of ovarian carcinoma. *Nature* **474**, 609–615 (2011). 10.1038/nature10166 PMC3163504 21720365

[5] Garsed, D. W. et al. Homologous recombination DNA repair pathway disruption and retinoblastoma protein loss are associated with exceptional survival in high-grade serous ovarian cancer. *Clin. Cancer Res.* **24**, 569–580 (2018). 29061645 10.1158/1078-0432.CCR-17-1621

[6] Muaibati, M. et al. Efficacy of immune checkpoint inhibitor monotherapy or combined with other small molecule-targeted agents in ovarian cancer. *Expert Rev. Mol. Med.* **25**, e6 (2023). 36691778 10.1017/erm.2023.3

[7] Hornburg, M. et al. Single-cell dissection of cellular components and interactions shaping the tumor immune phenotypes in ovarian cancer. *Cancer Cell* **39**, 928–944.e926 (2021). 33961783 10.1016/j.ccell.2021.04.004

[8] Kandalaft, L. E., Dangaj Laniti, D. & Coukos, G. Immunobiology of high-grade serous ovarian cancer: lessons for clinical translation. *Nat. Rev. Cancer* **22**, 640–656 (2022). 36109621 10.1038/s41568-022-00503-z

[9] Tothill, R. W. et al. Novel molecular subtypes of serous and endometrioid ovarian cancer linked to clinical outcome. *Clin. Cancer Res.* **14**, 5198–5208 (2008). 18698038 10.1158/1078-0432.CCR-08-0196

[10] Helland, A. et al. Deregulation of MYCN, LIN28B and LET7 in a molecular subtype of aggressive high-grade serous ovarian cancers. *PLoS ONE* **6**, e18064 (2011). 21533284 10.1371/journal.pone.0018064 PMC3076323

[11] Jimenez-Sanchez, A. et al. Unraveling tumor-immune heterogeneity in advanced ovarian cancer uncovers immunogenic effect of chemotherapy. *Nat. Genet* **52**, 582–593 (2020). 32483290 10.1038/s41588-020-0630-5 PMC8353209

[12] Gebauer, F., Schwarzl, T., Valcarcel, J. & Hentze, M. W. RNA-binding proteins in human genetic disease. *Nat. Rev. Genet* **22**, 185–198 (2021). 33235359 10.1038/s41576-020-00302-y

[13] Pereira, B., Billaud, M. & Almeida, R. RNA-binding proteins in cancer: old players and new actors. *Trends Cancer* **3**, 506–528 (2017). 28718405 10.1016/j.trecan.2017.05.003

[14] Turner, M. & Diaz-Munoz, M. D. RNA-binding proteins control gene expression and cell fate in the immune system. *Nat. Immunol.* **19**, 120–129 (2018). 29348497 10.1038/s41590-017-0028-4

[15] Nussinov, R., Yavuz, B. R. & Jang, H. Molecular principles underlying aggressive cancers. *Signal Transduct. Target Ther.* **10**, 42 (2025). 39956859 10.1038/s41392-025-02129-7 PMC11830828

[16] Bertoldo, J. B., Muller, S. & Huttelmaier, S. RNA-binding proteins in cancer drug discovery. *Drug Discov. Today* **28**, 103580 (2023). 37031812 10.1016/j.drudis.2023.103580

[17] Handley, K. F. et al. Classification of high-grade serous ovarian cancer using tumor morphologic characteristics. *JAMA Netw. Open* **5**, e2236626 (2022). 36239936 10.1001/jamanetworkopen.2022.36626 PMC9568802

[18] Schott, A. et al. The IGF2BP1 oncogene is a druggable m(6)A-dependent enhancer of YAP1-driven gene expression in ovarian cancer. *NAR Cancer* **7**, zcaf006 (2025). 40008228 10.1093/narcan/zcaf006 PMC11850222

[19] Gu, Y. et al. The biological roles of CD24 in ovarian cancer: old story, but new tales. *Front Immunol.* **14**, 1183285 (2023). 37359556 10.3389/fimmu.2023.1183285 PMC10288981

[20] Nguyen, T. M., Ngoc, D. T. M., Choi, J. H. & Lee, C. H. Unveiling the neural environment in cancer: exploring the role of neural circuit players and potential therapeutic strategies. *Cells* **12**10.3390/cells12151996 (2023). 10.3390/cells12151996 PMC10417274 37566075

[21] Guo, X. et al. Global characterization of T cells in non-small-cell lung cancer by single-cell sequencing. *Nat. Med.* **24**, 978–985 (2018). 29942094 10.1038/s41591-018-0045-3

[22] Gerstberger, S., Hafner, M. & Tuschl, T. A census of human RNA-binding proteins. *Nat. Rev. Genet* **15**, 829–845 (2014). 25365966 10.1038/nrg3813 PMC11148870

[23] Liu, Y. et al. Allosteric regulation of IGF2BP1 as a novel strategy for the activation of tumor immune microenvironment. *ACS Cent. Sci.* **8**, 1102–1115 (2022). 36032766 10.1021/acscentsci.2c00107 PMC9413439

[24] Bley, N. et al. Musashi-1-A Stemness RBP for cancer therapy? *Biology* **10**10.3390/biology10050407 (2021). 10.3390/biology10050407 PMC8148009 34062997

[25] Wan, W. et al. METTL3/IGF2BP3 axis inhibits tumor immune surveillance by upregulating N(6)-methyladenosine modification of PD-L1 mRNA in breast cancer. *Mol. Cancer* **21**, 60 (2022). 35197058 10.1186/s12943-021-01447-y PMC8864846

[26] Lin, X. et al. RNA-binding protein LIN28B inhibits apoptosis through regulation of the AKT2/FOXO3A/BIM axis in ovarian cancer cells. *Signal Transduct. Target Ther.* **3**, 23 (2018). 30174831 10.1038/s41392-018-0026-5 PMC6117292

[27] Elcheva, I. A. et al. IGF2BP family of RNA-binding proteins regulate innate and adaptive immune responses in cancer cells and tumor microenvironment. *Front. Immunol.* **14**10.3389/fimmu.2023.1224516 (2023). 10.3389/fimmu.2023.1224516 PMC10369348 37503349

[28] Patra, T. et al. Targeting Lin28 axis enhances glypican-3-CAR T cell efficacy against hepatic tumor initiating cell population. *Mol. Ther.* **31**, 715–728 (2023). 36609146 10.1016/j.ymthe.2023.01.002 PMC10014222

[29] Feng, T. et al. DEAD-Box Helicase DDX25 is a negative regulator of Type I interferon pathway and facilitates RNA virus infection. *Front. Cell Infect. Microbiol.* **7**, 356 (2017). 28824886 10.3389/fcimb.2017.00356 PMC5543031

[30] Xie, Q. et al. Activation of insulin-like growth factor-1 receptor (IGF-1R) promotes growth of colorectal cancer through triggering the MEX3A-mediated degradation of RIG-I. *Acta Pharm. Sin. B* **13**, 2963–2975 (2023). 37521868 10.1016/j.apsb.2023.04.001 PMC10372823

[31] Matsumoto, T. et al. Anaplastic Lymphoma kinase overexpression is associated with aggressive phenotypic characteristics of ovarian high-grade serous carcinoma. *Am. J. Pathol.* **191**, 1837–1850 (2021). 34214505 10.1016/j.ajpath.2021.06.009

[32] Domcke, S., Sinha, R., Levine, D. A., Sander, C. & Schultz, N. Evaluating cell lines as tumour models by comparison of genomic profiles. *Nat. Commun.* **4**, 2126 (2013). 23839242 10.1038/ncomms3126 PMC3715866

[33] Dutil, J., Chen, Z., Monteiro, A. N., Teer, J. K. & Eschrich, S. A. An interactive resource to probe genetic diversity and estimated ancestry in cancer cell lines. *Cancer Res.* **79**, 1263–1273 (2019). 30894373 10.1158/0008-5472.CAN-18-2747 PMC6445675

[34] Kwok, A. L. et al. Caution over use of ES2 as a model of ovarian clear cell carcinoma. *J. Clin. Pathol.* **67**, 921–922 (2014). 25049276 10.1136/jclinpath-2014-202430

[35] Hannus, M. et al. siPools: highly complex but accurately defined siRNA pools eliminate off-target effects. *Nucleic Acids Res.* **42**, 8049–8061 (2014). 24875475 10.1093/nar/gku480 PMC4081087

[36] Walton, J. et al. CRISPR/Cas9-mediated Trp53 and Brca2 knockout to generate improved murine models of ovarian high-grade serous carcinoma. *Cancer Res.* **76**, 6118–6129 (2016). 27530326 10.1158/0008-5472.CAN-16-1272 PMC5802386

[37] Muller, S. et al. The oncofetal RNA-binding protein IGF2BP1 is a druggable, post-transcriptional super-enhancer of E2F-driven gene expression in cancer. *Nucleic Acids Res.* **48**, 8576–8590 (2020). 32761127 10.1093/nar/gkaa653 PMC7470957

[38] Muller, S. et al. IGF2BP1 enhances an aggressive tumor cell phenotype by impairing miRNA-directed downregulation of oncogenic factors. *Nucleic Acids Res.* **46**, 6285–6303 (2018). 29660014 10.1093/nar/gky229 PMC6158595

[39] Hagemann, S. et al. IGF2BP1 induces neuroblastoma via a druggable feedforward loop with MYCN promoting 17q oncogene expression. *Mol. Cancer* **22**, 88 (2023). 37246217 10.1186/s12943-023-01792-0 PMC10226260

[40] Huang, H. et al. Recognition of RNA N(6)-methyladenosine by IGF2BP proteins enhances mRNA stability and translation. *Nat. Cell Biol.* **20**, 285–295 (2018). 29476152 10.1038/s41556-018-0045-z PMC5826585

[41] Ni, Z. et al. JNK signaling promotes bladder cancer immune escape by regulating METTL3-mediated m6A modification of PD-L1 mRNA. *Cancer Res.* **82**, 1789–1802 (2022). 35502544 10.1158/0008-5472.CAN-21-1323

[42] Landre, V., Pion, E., Narayan, V., Xirodimas, D. P. & Ball, K. L. DNA-binding regulates site-specific ubiquitination of IRF-1. *Biochem. J.* **449**, 707–717 (2013). 23134341 10.1042/BJ20121076

[43] Audrito, V. et al. PD-L1 up-regulation in melanoma increases disease aggressiveness and is mediated through miR-17-5p. *Oncotarget* **8**, 15894–15911 (2017). 28199980 10.18632/oncotarget.15213 PMC5362532

[44] Liu, S. et al. CD4(+)CCR8(+) Tregs in ovarian cancer: a potential effector Tregs for immune regulation. *J. Transl. Med.* **21**, 803 (2023). 37950246 10.1186/s12967-023-04686-3 PMC10638792

[45] Chou, T. C. Drug combination studies and their synergy quantification using the Chou-Talalay method. *Cancer Res.* **70**, 440–446 (2010). 20068163 10.1158/0008-5472.CAN-09-1947

[46] Zhao, Y. et al. Cytotoxicity enhancement in MDA-MB-231 cells by the combination treatment of tetrahydropalmatine and berberine derived from Corydalis yanhusuo W. T. Wang. *J. Intercult. Ethnopharmacol.* **3**, 68–72 (2014). 26401350 10.5455/jice.20140123040224 PMC4576799

[47] Taylor, B. C. & Balko, J. M. Mechanisms of MHC-I downregulation and role in immunotherapy response. *Front. Immunol.* **13**, 844866 (2022). 35296095 10.3389/fimmu.2022.844866 PMC8920040

[48] Bley, N. et al. IGF2BP1 is a targetable SRC/MAPK-dependent driver of invasive growth in ovarian cancer. *RNA Biol.* **18**, 391–403 (2021). 32876513 10.1080/15476286.2020.1812894 PMC7951963

[49] Muller, S. et al. IGF2BP1 promotes SRF-dependent transcription in cancer in a m6A- and miRNA-dependent manner. *Nucleic Acids Res.* **47**, 375–390 (2019). 30371874 10.1093/nar/gky1012 PMC6326824

[50] Mu, H. et al. RNA binding protein IGF2BP1 meditates oxidative stress-induced granulosa cell dysfunction by regulating MDM2 mRNA stability in an m(6)A-dependent manner. *Redox Biol.* **57**, 102492 (2022). 36182806 10.1016/j.redox.2022.102492 PMC9526231

[51] Mahapatra, L., Andruska, N., Mao, C., Le, J. & Shapiro, D. J. A Novel IMP1 Inhibitor, BTYNB, Targets c-Myc and Inhibits Melanoma and Ovarian Cancer Cell Proliferation. *Transl. Oncol.* **10**, 818–827 (2017). 28846937 10.1016/j.tranon.2017.07.008 PMC5576976

[52] Nie, W. et al. Cucurbitacin B and its derivatives: a review of progress in biological activities. *Molecules* **29**10.3390/molecules29174193 (2024). 10.3390/molecules29174193 PMC11397067 39275042

[53] Gupta, H. B. et al. Tumor cell-intrinsic PD-L1 promotes tumor-initiating cell generation and functions in melanoma and ovarian cancer. *Signal Transduct. Target Ther.* **1**, 16030 (2016). 28798885 10.1038/sigtrans.2016.30 PMC5547561

[54] Murthy, D. et al. CD24 negativity reprograms mitochondrial metabolism to PPARalpha and NF-kappaB-driven fatty acid beta-oxidation in triple-negative breast cancer. *Cancer Lett.* **587**, 216724 (2024). 38373689 10.1016/j.canlet.2024.216724 PMC11068061

[55] Zhong, G., Chang, X., Xie, W. & Zhou, X. Targeted protein degradation: advances in drug discovery and clinical practice. *Signal Transduct. Target Ther.* **9**, 308 (2024). 39500878 10.1038/s41392-024-02004-x PMC11539257

[56] Wang, Y. et al. Anti-PD-1 antibody armored gammadelta T cells enhance anti-tumor efficacy in ovarian cancer. *Signal Transduct. Target Ther.* **8**, 399 (2023). 37857598 10.1038/s41392-023-01646-7 PMC10587135

[57] Justin C. Moser, K. P. P., Jordi Rodon Ahnert, Yaara Ofir-Rosenfeld, and Josefin-Beate Holz. Phase 1 dose escalation and cohort expansion study evaluating safety, PK, PD and clinical activity of STC-15, a METTL-3 inhibitor, in patients with advanced malignancies. *J. Clin. Oncol.* **42**10.1200/JCO.2024.42.16_suppl.2586 (2024).

[58] Busch, B. et al. The oncogenic triangle of HMGA2, LIN28B and IGF2BP1 antagonizes tumor-suppressive actions of the let-7 family. *Nucleic Acids Res.* **44**, 3845–3864 (2016). 26917013 10.1093/nar/gkw099 PMC4856984

[59] Galaxy, C. The Galaxy platform for accessible, reproducible and collaborative biomedical analyses: 2022 update. *Nucleic Acids Res.* **50**, W345–W351 (2022). 35446428 10.1093/nar/gkac247 PMC9252830

[60] Robinson, M. D., McCarthy, D. J. & Smyth, G. K. edgeR: a Bioconductor package for differential expression analysis of digital gene expression data. *Bioinformatics* **26**, 139–140 (2010). 19910308 10.1093/bioinformatics/btp616 PMC2796818

[61] Castanza, A. S. et al. Extending support for mouse data in the Molecular Signatures Database (MSigDB). *Nat. Methods* **20**, 1619–1620 (2023). 37704782 10.1038/s41592-023-02014-7 PMC11397807

[62] Germain, P. L., Lun, A., Garcia Meixide, C., Macnair, W. & Robinson, M. D. Doublet identification in single-cell sequencing data using scDblFinder. *F1000Res* **10**, 979 (2021). 35814628 10.12688/f1000research.73600.1 PMC9204188

[63] Hao, Y. et al. Integrated analysis of multimodal single-cell data. *Cell* **184**, 3573–3587.e3529 (2021). 34062119 10.1016/j.cell.2021.04.048 PMC8238499

[64] Rath, S. et al. MitoCarta3.0: an updated mitochondrial proteome now with sub-organelle localization and pathway annotations. *Nucleic Acids Res.* **49**, D1541–D1547 (2021). 33174596 10.1093/nar/gkaa1011 PMC7778944

[65] Nosova, E. V. et al. V.N. Synthesis and photophysical studies of novel 2-[5-(4-diethylaminophenyl)thiophen-2-yl]quinazoline derivatives. Mendeleev Commun. **28**, 14–16 (2018).

[66] Bankhead, P. et al. QuPath: Open source software for digital pathology image analysis. *Sci. Rep.* **7**, 16878 (2017). 29203879 10.1038/s41598-017-17204-5 PMC5715110

