# Supplementary material for: Inhibition of RNA-binding proteins enhances immunotherapy in ovarian cancer
Source: Signal Transduct Target Ther. 2025 Dec 25;10:419. doi: 10.1038/s41392-025-02515-1 (PMC12739169; doi:10.1038/s41392-025-02515-1)
Supplement: Supplementary file 1 — Supplementary Material [file 41392_2025_2515_MOESM1_ESM.docx]

Supplementary Materials for

**Inhibition of RNA-binding proteins enhances immunotherapy in ovarian cancer**

Nadine Bley, Alexander Rausch, Simon Müller, Theresa Simon, Markus Glaß, Danny Misiak, Laura Schian, Lara Meret Peters, Mohammad Dipto, Ali Hmedat, Bianca Busch, Annekatrin Schott, Marcell Lederer, Alice Wedler, Robin Benedikt Rolnik, Hend Elrewany, Ehab Ghazy, Wolfgang Sippl, Martina Vetter, Markus Wallwiener, and Stefan Hüttelmaier

Correspondence to: [nadine.stoehr@medizin.uni-halle.de](mailto:nadine.stoehr@medizin.uni-halle.de)

**This PDF file includes:**

Supplementary Fig. 1

Supplementary Fig. 2

Supplementary Fig. 3

Supplementary Fig. 4

Supplementary Fig. 5

Supplementary Fig. 6

Supplementary Fig. 7

Supplementary Fig. 8

Supplementary Fig. 9

Supplementary Fig. 10

**Supplementary Fig. S1.** C5-tumors show low immune cell infiltration and high RBP expression.


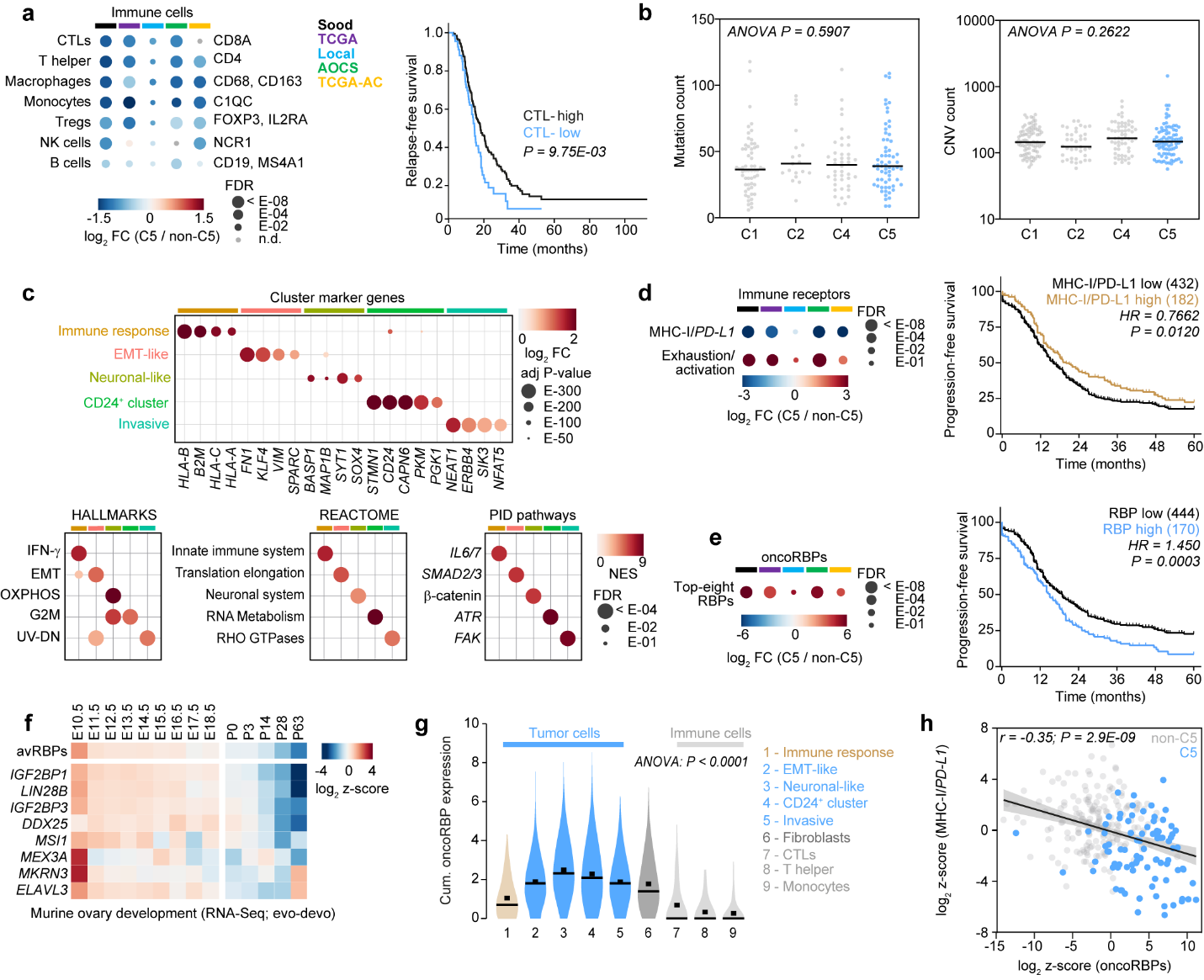


**a,** Changes in immune cell infiltration associated with C5 tumors are shown as a bubble chart depicting the indicated immune cell types by respective marker genes. The impact of CTLs on relapse-free survival is presented in a Kaplan-Meier plot generated using [GEPIA2021](http://gepia2021.cancer-pku.cn/) and CIBERSORT deconvolution. Among the immune cell types analyzed, only CTLs demonstrated prognostic significance. **b,** Scatter plots summarizing the number of mutations and copy number variations (CNVs) per patient in the TCGA-RNASeq cohort. Data were obtained from [cBioPortal](https://www.cbioportal.org/). Statisticcal significance was determined by a One-way-Anova. **c,** Marker genes for tumor cell clusters (upper panel), as determined by scRNA-seq, and GSEA-based cluster characteristics (lower panels) are presented as a bubble charts. Gene ranking was performed based on marker gene expression, followed by GSEA analysis. Log_2_ fold-changes (log_2_FC) or normalized enrichment scores (NES) are indicated by color. **d, e,** Bubble chart representations of immune receptor (d) or oncoRBP expression (e) in C5 vs. non-C5 tumors of indicated datasets using the following markers for T cell exhaustion: *CXCL13*, *HAVCR2*, *LAG3*, *LAYN*, *PDCD1*, *PRDM1*, *TIGIT*; T cell activation: *CD69*, *GZMA*, *GZMB*, *HLA-DRA*, *HLA-DRB1*, *IFNG*, *TNF*; and oncoRBPs: *DDX25*, *ELAVL3*, *IGF2BP1*, *IGF2BP3*, *LIN28B*, *MEX3A*, *MKRN3* and *MSI1*. Kaplan-Meier plots were generated using [KMplot](http://www.kmplot.com). The hazard ratio (HR), the number of patients in each group and the p-value determined by log-rank testing is indicated in the graph. **f,** Heatmap displaying gene expression levels of selected RBPs during murine ovary development by [evo-devo](https://apps.kaessmannlab.org/evodevo/). **g,** Cumulative scRNA-seq reads of the top eight oncoRBPs for indicated tumor and stroma cell clusters. Tumor cell clusters with high RBP expression are highlighted in blue. **h,** Pearson correlation analysis of MHC-I/*PD-L1* ratios versus oncoRBP z-scores indicates a negative association of both measures. C5 tumors are highlighted in blue.

**Supplementary Fig. S2.** OncoRBPs like *IGF2BP1* promote immune evasion.


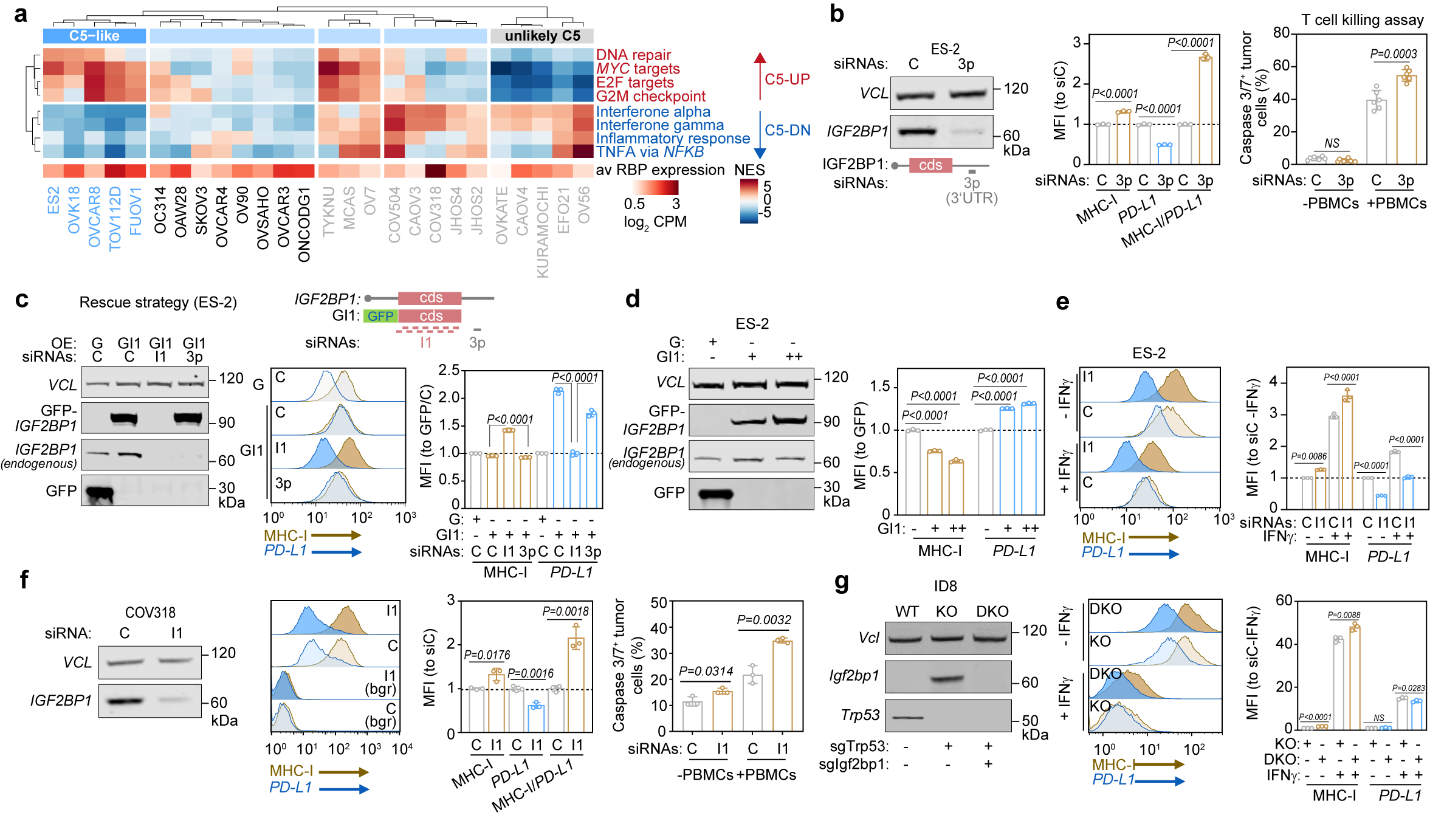


**a,** Classification of *TP53*-mutated ovarian cancer cell lines using GSEA. Gene expression was ranked by log_2_ FC over the median (CCLE RNA-Seq dataset). NES scores of hallmark gene sets upregulated (C5-UP, red) or downregulated (C5-DN, blue) in C5 tumors are shown alongside oncoRBP expression as a clustered heatmap. **b,** *IGF2BP1* knockdown in ES-2 cells using an alternative siRNA directed against the 3’UTR (3p) or control siRNAs (C). Knockdown was analyzed by Western blotting with indicated antibodies (left). *VCL* served as loading control. Flow cytometry analyses for MHC-I and *PD-L1* presentation were used to determine mean fluorescence intensities (MFI) relative to the control transfection (middle). T cell-mediated tumor cell killing was determined by caspase-3/7 activity upon co-culture with PBMCs using an Incucyte S3 device (right). **c,** Rescue experiment using GFP (G) or GFP-*IGF2BP1* (GI1) overexpressing ES-2 cells transfected with control siRNAs (C), an *IGF2BP1*-targeting siRNA pool (I1, 12 siRNAs) or the alternative siRNA directed against the 3’UTR (3p). The rescue strategy is depicted above the panel. Knockdown efficiencies and MHC-I/*PD-L1* presentation were analyzed as in (b). **d,** Dose-dependent IGF2BP1 overexpression was analyzed by Western blot and flow cytometry as in (b). **e,** siRNA-pool mediated knockdown of *IGF2BP1* (I1) treated with IFNγ (100 ng/mL) for 48 h was analyzed by flow cytometry as in (b). **f,** IGF2BP1 depletion by siRNA pools was analyzed in COV318 as in (b). **g,** Wildtype (WT) murine ID8 ovarian cells, *Trp53* knockout (KO) and *Trp53/Igf2bp1* double knockout (DKO) cells were analyzed by Western blot and flow cytometry with or without IFNγ treatment (100 ng/mL for 48 h) as indicated. Error bars indicate s.d. from N > 3 experiments. Statistical significance was determined by an unpaired two-tailed t test.

**Supplementary Fig. S3.** *IGF2BP1* promotes immune evasion in syngeneic mouse models.

**
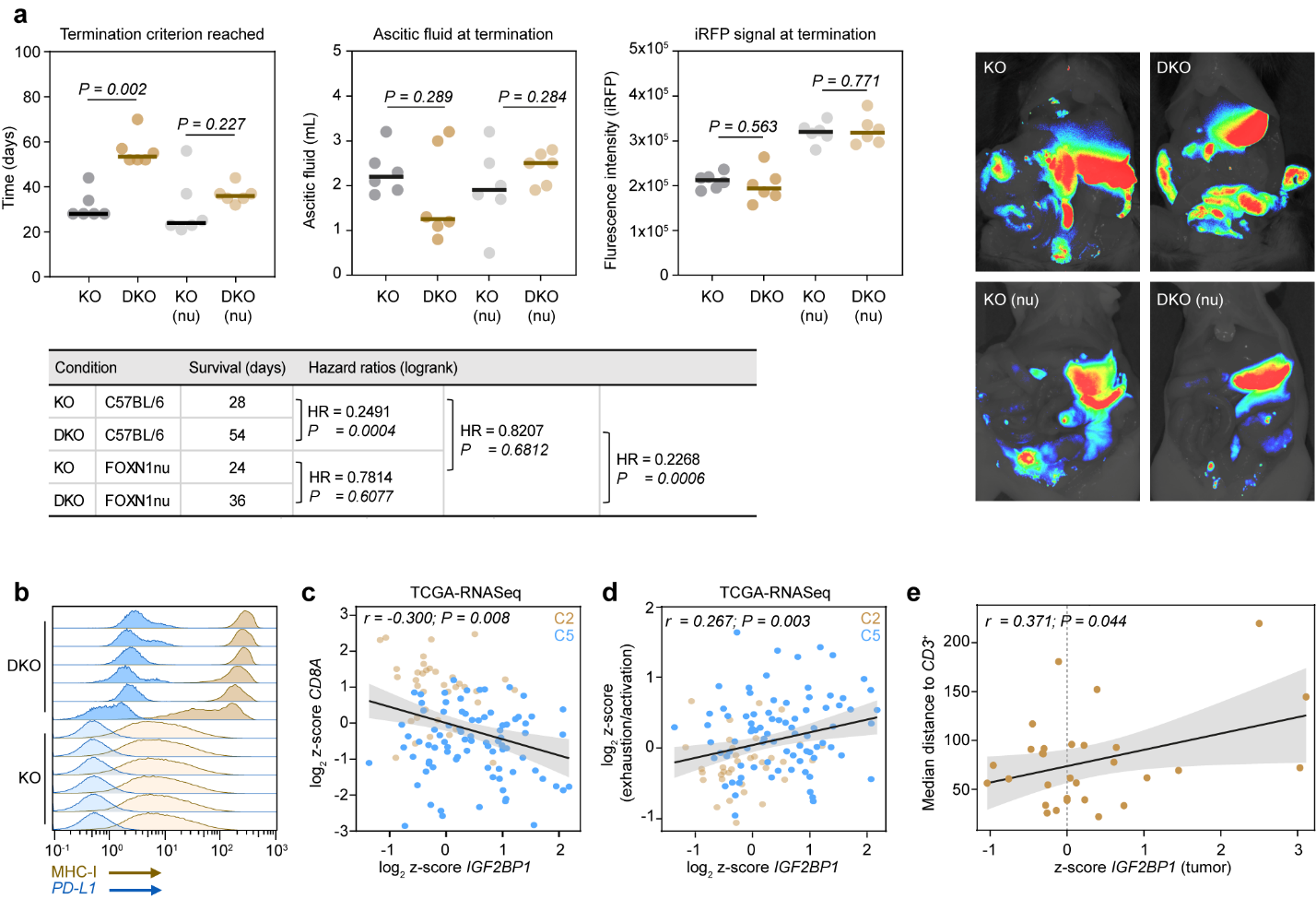
**

**a,** Syngeneic mouse model using intraperitoneal injection of iRFP-labeled ID8-*Trp53/Igf2bp1*-DKO and ID8-*Trp53*-KO cells in immunocompetent or immunodeficient mice. Time of termination (left), ascitic fluid volume at the time of termination (middle) and tumor burden at the time of termination, measured by iRFP fluorescence intensity (right), were analyzed using an unpaired two-tailed t test and presented as scatter dot plots. Representative iRFP images are shown (left). The table indicates hazard ratios, p-values determined by logrank testing, and median survival times for the analyzed animal cohorts using 6 mice per condition. **b,** Histograms from flow cytometry analyses of ascitic fluid show a marked increase in MHC-I and a moderate increase in *PD-L1* expression on iRFP-positive ID8-*Trp53/Igf2bp1*-DKO cells compared to ID8-Trp53-KO cells. **c, d,** Pearson correlation analyses between *IGF2BP1* expression and CTL abundance (*CD8A*, left) or the ratio of exhaustion (*CXCL13, HAVCR2, LAG3, LAYN, PDCD1, PRDM1, TIGIT*) to activation (*CD69, GZMA, GZMB, HLA-DRA, HLA-DRB1, IFNG, TNF*) markers (right) using C2 and C5 classified samples from the TCGA-RNASeq cohort. **e,** Multispectral imaging of a human HGSC-TMA using the Opal-7 system. Single-cell segmentation and classification were performed using QuPath. Pearson correlation analysis was applied to evaluate the relationship between *IGF2BP1*-expressing, cytokeratin-positive tumor cells and their mean distance of *CD3*^+^ T cells.

**Supplementary Fig. S4.** *IGF2BP1* enhances *IRF1* protein decay by stabilizing *MDM2* mRNA.

**
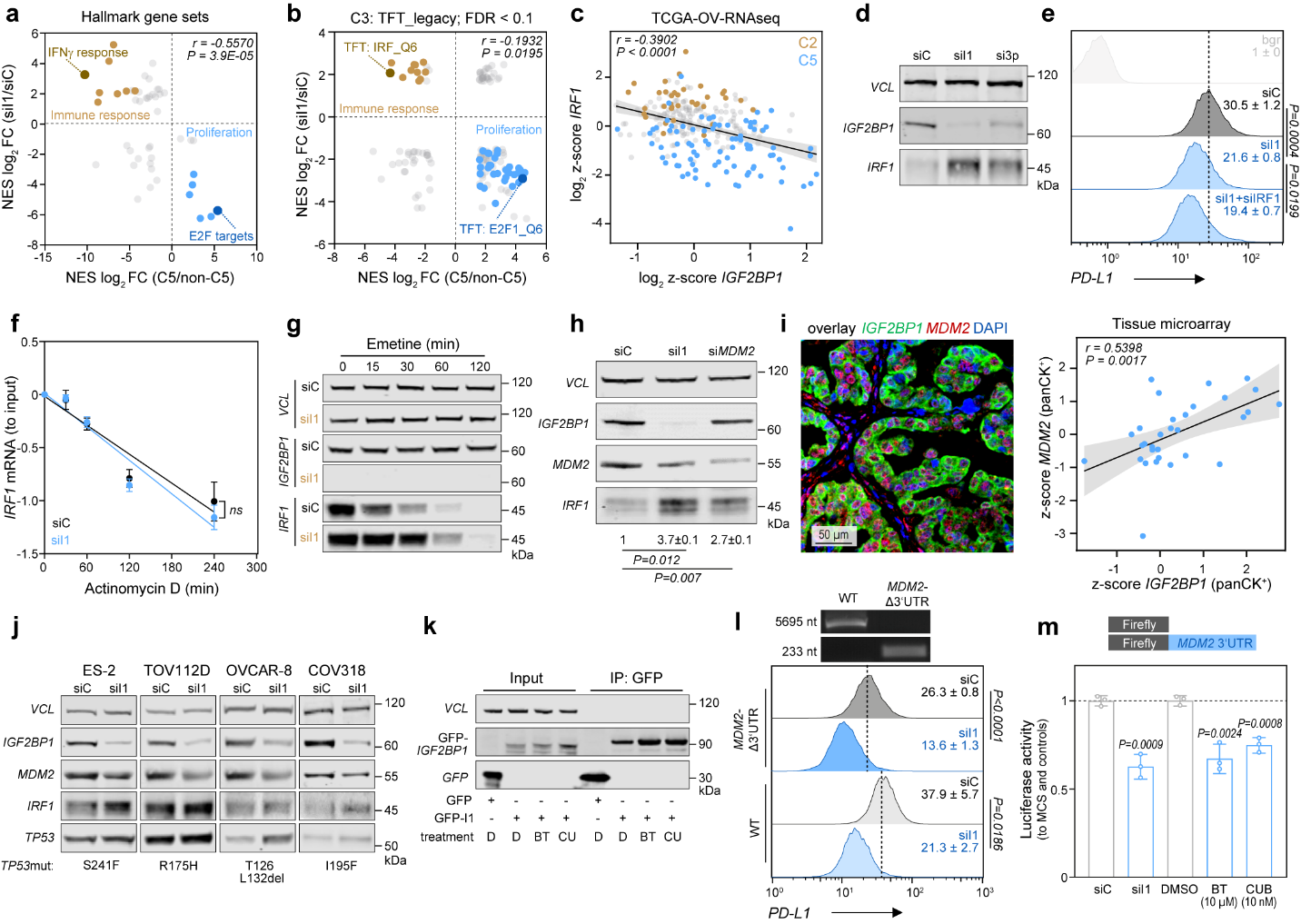
**

**a, b,** C5 tumors and *IGF2BP1*-depleted cells show inversed enrichment of Hallmark gene sets (a) and C3-TFT gene sets (b). **c,** Pearson correlation determined a negative association of *IGF2BP1* and *IRF1* mRNAs (TCGA-RNASeq cohort). C2 and C5 tumors are indicated by color. **d,** Changes in *IRF1* expression upon *IGF2BP1* knockdown using the pool of 12 siRNAs (siI1) or the alternative 3’UTR-directed siRNA (3p) in ES-2 cells were analyzed by Western blotting with indicated antibodies and *VCL* as loading control. **e,** Phenotype rescue experiment of *IGF2BP1* depletion and co-depletion of *IRF1* in ES-2 cells analyzed by flow cytometry for *PD-L1* presentation as shown by histograms. Numbers indicate MFI and s.d. (N = 3; unpaired two-tailed t test). **f,** *IRF1* mRNA turnover was monitored upon actinomycin D treatment (5 µM). Chi sqr comparison reveal no statistical significance (ns). **g,** *IRF1* protein turnover was monitored upon emetine treatment (100 µM) to block translation. Quantifications are shown in Fig. 4c. **h,** *IGF2BP1* and *MDM2* knockdowns in ES-2 cells were analyzed by Western blotting with indicated antibodies as in d. Quantifications indicate average and s.d (N = 2; unpaired two-tailed t test). **i,** Pearson correlation indicates co-expression of *IGF2BP1* and *MDM2* proteins in panCK-positive tumor cells by multi-spectral imaging (right). A representative overlay image is shown for indicated stanings (left). **j,** *IGF2BP1* knockdown in indicated cell lines was analyzed as in (d). *TP53* mutations are indicated below panel. **k,** RNA-immunoprecipitation (RIP) using GFP-directed antibodies in ES-2 with *IGF2BP1* deletion and re-expression of GFP-*IGF2BP1*. GFP served as negative control. Cells were treated with DMSO (D), BTYNB (BT; 10 µM) or Cucurbitacin B (CUB; 10 nM) 24 h before the RIP. Western blotting indicates equal protein precipitation. Associated transcripts are shown in Fig. 4e. **l,** The endogenous *MDM2*-3’UTR was deleted in ES-2 cells. Deletion was verified by genomic PCR (left). *PD-L1* presentation was determined by flow cytometry shown as histogram. Numbers indicate MFI and s.d. (N = 3; unpaired two-tailed t test). **m,** Luciferase reporter studies using the *MDM2*-3’UTR upon *IGF2BP1* depletion or inhibition. Statistical significance was determined by an unpaired two-tailed t test.

**Supplementary Fig. S5.** *IGF2BP1* promotes *PD-L1* expression in a miRNA-dependent manner.


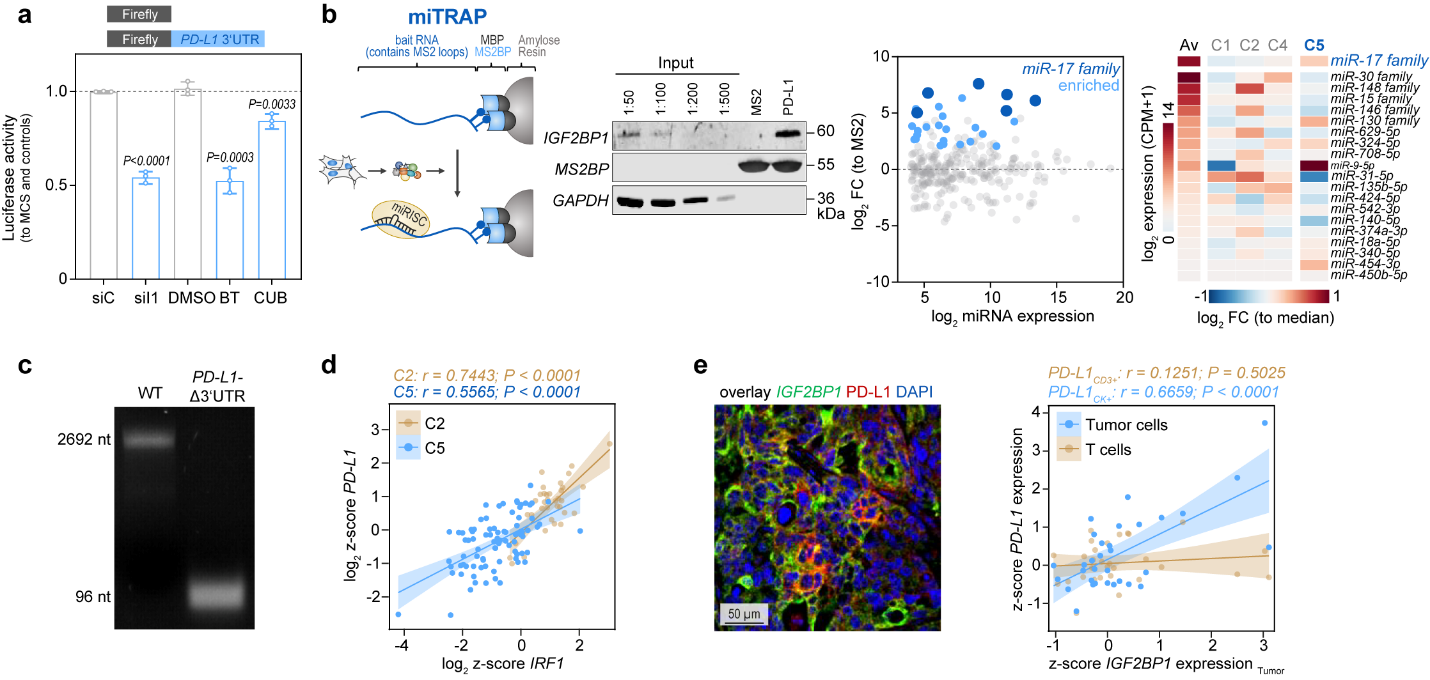


**a,** Luciferase reporter studies using the *PD-L1*-3’UTR upon *IGF2BP1* depletion (72 h) or inhibition by BT (10 µM) or CUB (10 nM) for 48 h in ES-2 cells. Statistical significance was determined by an unpaired two-tailed t test. **b,** miTRAP experiments were used to identify miRNA associated to the *PD-L1*-3’UTR. Schematic (left) depicts experimental work flow. Western blotting with indicated antibodies confirms *IGF2BP1* binding to the *PD-L1*-3’UTR (middle left). PD-L1-3’UTR-associated miRNAs from ES-2 cell extracts were identified by RNA sequencing. MA plot indicates miRNAs significantly enriched (FDR < 0.05; light blue) to the bead control (MS2) and input by color (middle right). The miR-17 seed family (*miR-17-5p, miR-20a-5p, miR-20b-5p, miR-93-5p, miR-106a-5p* and *miR-106b-5p*) showing the strongest enrichment is indicated in blue. Heatmap indicates average expression (AV) of miRNA seed families and their log_2_ FC over median in the respective HGSC subtypes (right; TCGA-RNASeq) that were enriched in *PD-L1*-3’UTR-miTRAP experiments. **c,** The endogenous *PD-L1*-3’UTR was deleted in ES-2 cells. Deletion was verified by genomic PCR. **d,** Pearson correlation of *IRF1* and *PD-L1* mRNAs (TCGA-RNASeq) in C2 (yellow) and C5 (blue) subtypes. **e,** *IGF2BP1* and *PD-L1* proteins are co-expressed in tumor cells. Tumor tissue was identified by cytokeratin staining and T cells were labeled with *CD3* antibodies using multi-spectral imaging by Opal-7 staining. Significant Pearson correlation between *IGF2BP1* and *PD-L1* was identified in tumor cells (blue), but not T cells (yellow).

**Supplementary Fig. S6.** *IGF2BP1* inhibition enhances the immune response and promotes immune checkpoint therapy *in vitro*.

**
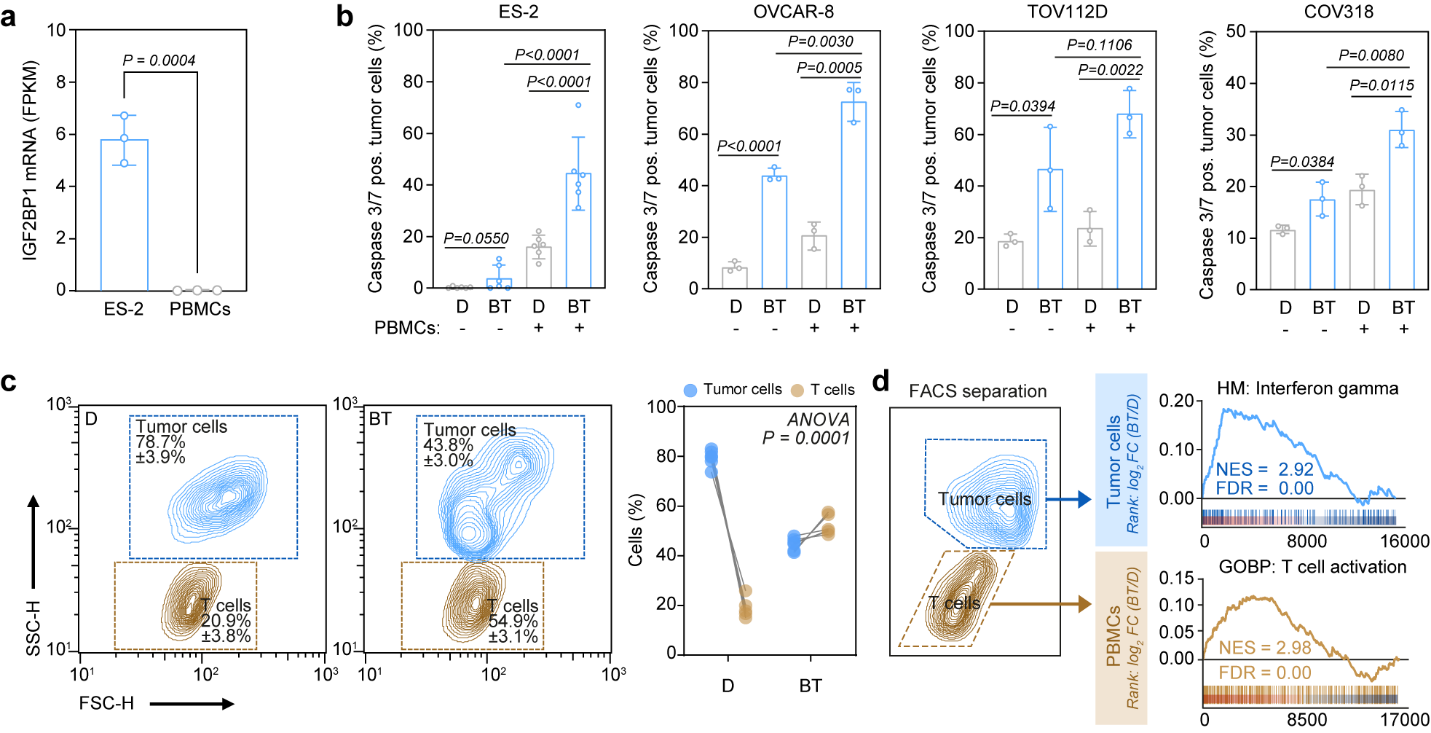
**

**a,** *IGF2BP1* mRNA expression of ES-2 cells and PBMCs was derived from polyA-RNA-seq experiments from monocultures. **b,** T cell-mediated tumor cell killing by determining caspase-3/7 activity upon coculture with HLA-matched PBMCs using an Incucyte S3 for indicated HGSC cell lines treated with BT (10 µM) or DMSO (D). Statistical significance (a, b) was determined by an unpaired two-tailed t test for N > 3 experiments. **c,** Flow cytometry analyses of cocultures of ES-2 cells and HLA-matched PBMCs treated with BT (10 µM) or DMSO as control. Contour plots indicate tumor (blue) and T cell (yellow) populations. The percentage of tumor and T cells for each condition is shown as matched pairs plot using a two-way ANOVA test to determine statistical significance. **d,** ES-2 cells and PBMCs from co-cultures with BT treatment (10 µM) were FACS separated before RNA extraction followed by polyA-RNA-seq. GSEA identified enrichments of indicated hallmark and GO-term biological process (GOBP) gene sets for the respective cell populations.

**Supplementary Fig. S7.** *IGF2BP1* inhibition enhances the immune response and promotes immune checkpoint therapy *in vivo*.


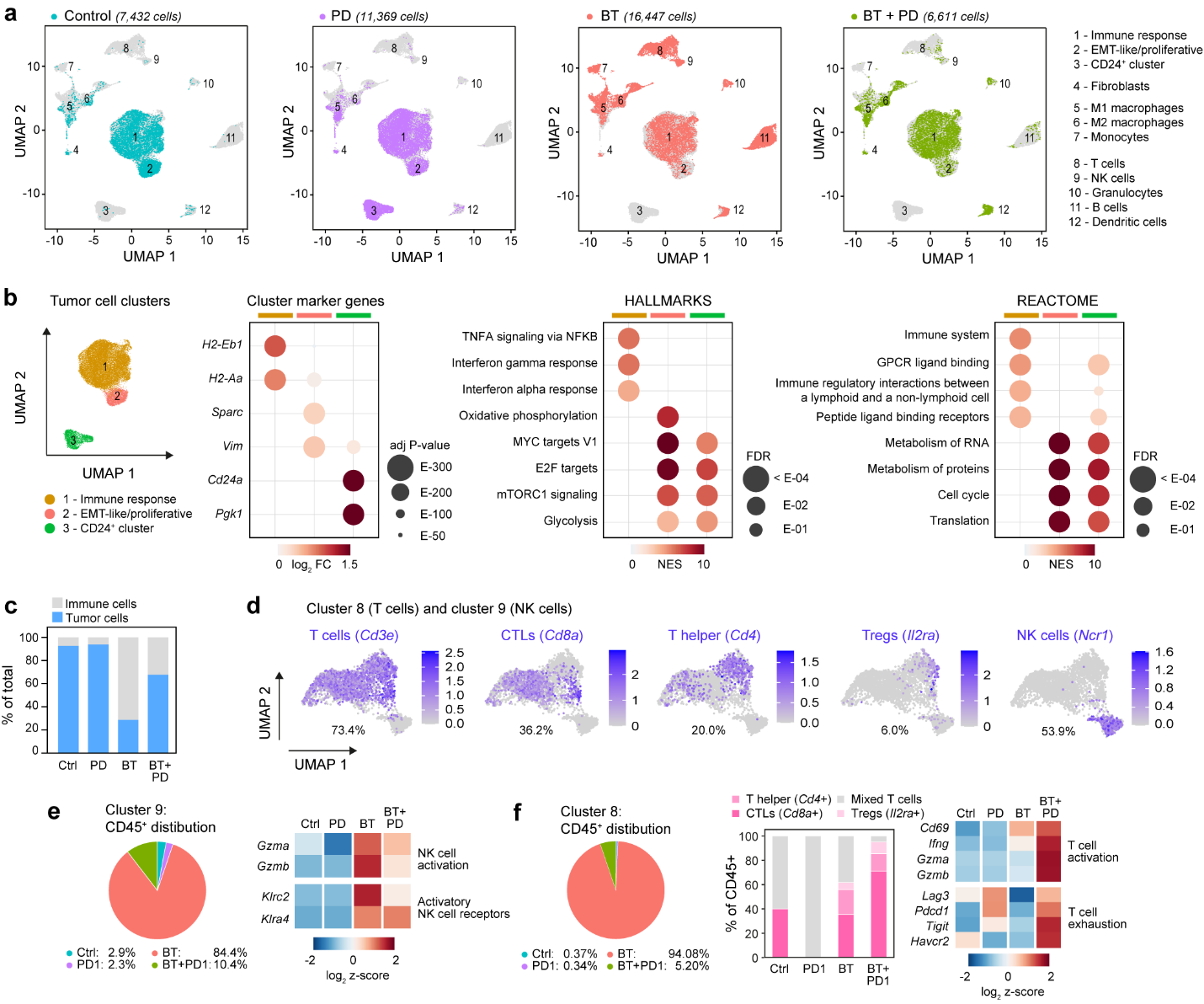


Ascites fluids from mice treated with BTYNB (BT), anti-PD-1 antibodies (PD), a combination of BT + anti-PD-1 antibodies (BT+PD), or vehicle/IgG2a (Control) were pooled per condition and analyzed by scRNA-seq. **a,** UMAP plots show distribution of tumor and immune cell clusters per treatment. Immune-responsive tumor cells (1) were present across all conditions, though at different frequencies. The Cd24⁺ cluster (3) was almost exclusively found upon anti-PD-1 monotherapy. **b,** Differential gene expression between the three tumor cell clusters (UMAP, left) was determined. Cluster-specific marker genes (middle left), shared with human tumors, are shown as bubble plot. Log₂ fold-change values were used to rank genes for hallmark (middle right) and Reactome (right) pathway enrichment (bubble plot). **c,** Bar graph summarizes proportions of tumor versus immune cells per treatment condition. **d,** Expression of canonical marker genes identifies T-cell subsets within cluster 8. Scale bars indicate normalized expression of subset-specific T and NK cell markers. Proportions indicate percent of Cd45^+^ cells. **e,** Pie chart shows distribution of Cd45⁺ lymphocytes in cluster 9 across treatments; frequencies are indicated below. Heatmap depicts average expression of activating NK-cell receptors and NK activation markers. **f,** Pie chart shows distribution of Cd45⁺ lymphocytes in cluster 8 across treatments; frequencies are indicated below. Bar graphs show distribution of T cell subsets within cluster 9 as percentage of Cd45⁺ cells per treatment. Heatmap depicts average expression of T cell activation and exhaustion markers across conditions.

**Supplementary Fig. S8.** BTYNB synthesis and purity.

BTYNB was synthesized as a beige solid (mp 174–176 °C; yield: 1.5 g, 70%). ^1H-NMR (400 MHz, DMSO-d₆): δ 8.49 (s, 1H), 7.61–7.57 (m, 1H), 7.31–7.21 (m, 2H), 7.06 (d, J = 3.8 Hz, 1H), 6.94 (d, J = 3.7 Hz, 1H), 6.69–6.74 (m, 2H), 5.94 (m, 1H). Purity was determined by HPLC (UV 254 nm) and found to be 95.9%.

**Supplementary Fig. S9.** Gating strategies.


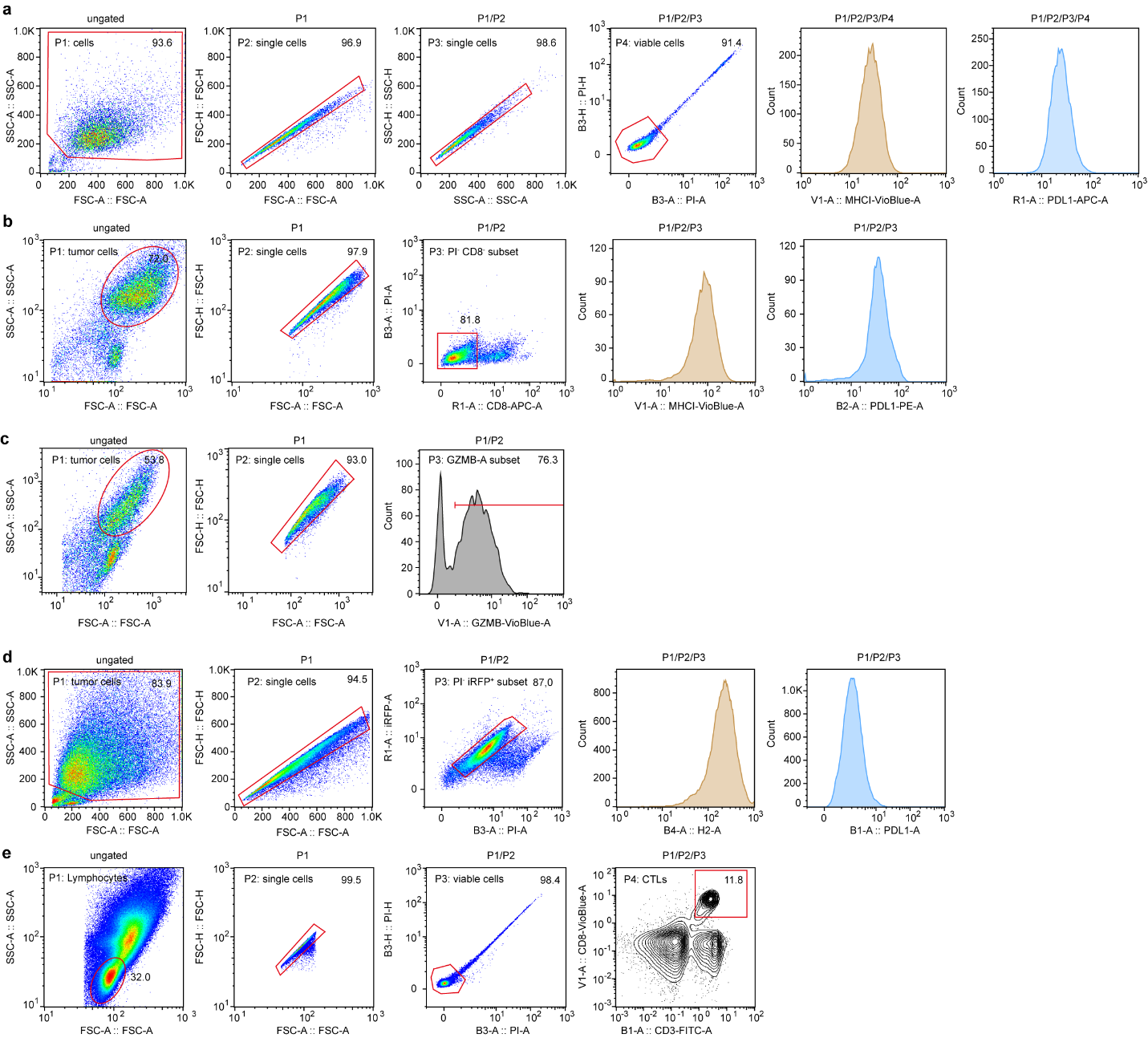


Gating strategies used to determine the following parameters are shown. **a**, MHC-I and PD-L1 expression on tumor cells (corresponding to Figures 2b, 4b, 4f, 5e, S2b–g, S4e, and S4l). **b**, MHC-I and PD-L1 expression on tumor cells from cocultures with PBMCs (Figures 2c and 6f). **c**, Intracellular GZMB in tumor cells (Figures 2e and 6g). **d**, MHC-I (H2) and PD-L1 expression on tumor cells from ascitic fluids (Figures 3b and S3b). **e**, CTLs identified as CD8⁺/CD3⁺ cells from ascitic fluids (Figure 3b).

**Supplementary Fig. S10.** Western blots.


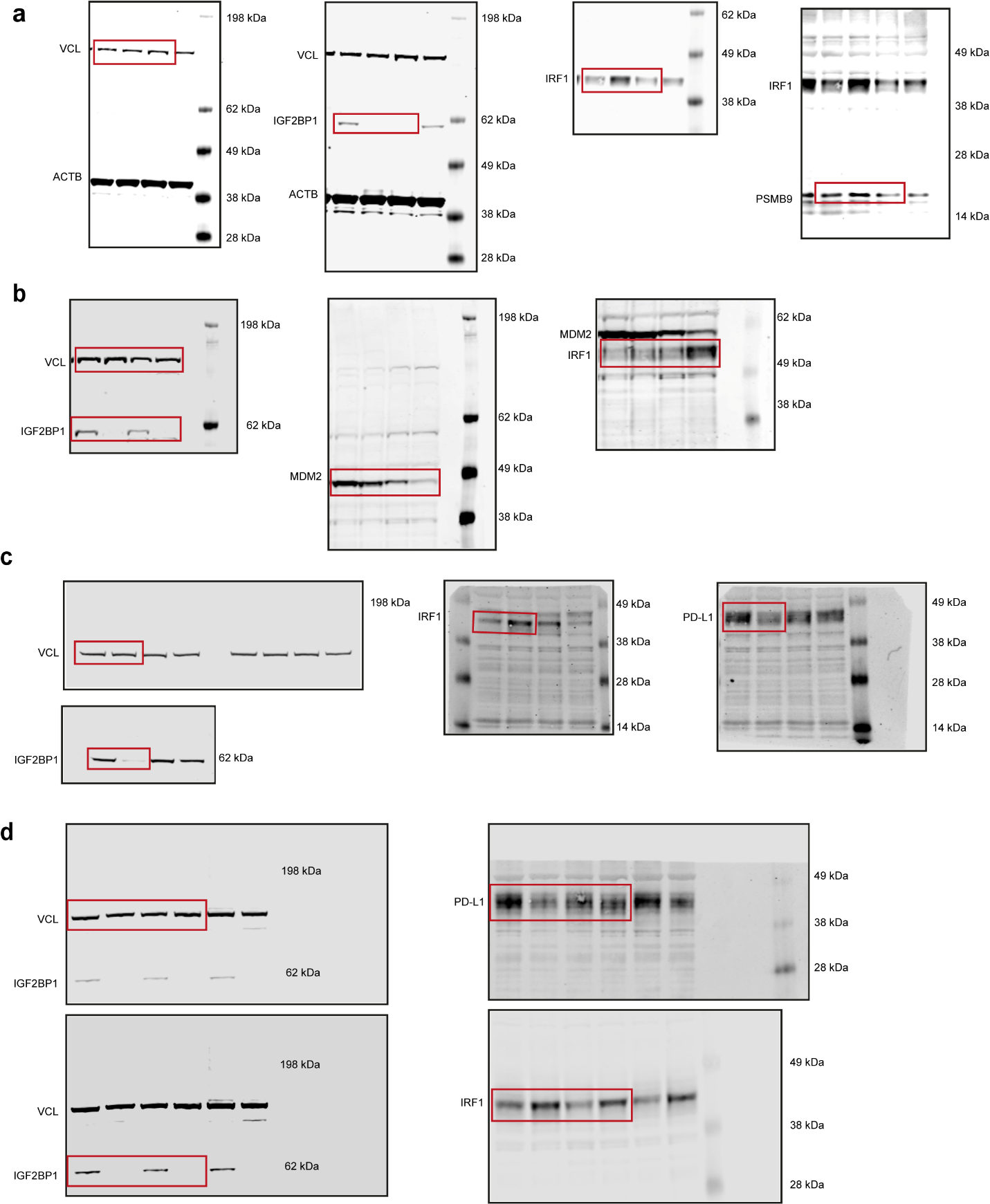


Full scans of Western blots corresponding to a, Figure 4b; b, Figure 4f; c, Figure 5b; and d, Figure 5e.
